# Monogamy promotes altruistic sterility in insect societies

**DOI:** 10.1098/rsos.172190

**Published:** 2018-05-09

**Authors:** Nicholas G. Davies, Andy Gardner

**Affiliations:** 1 London School of Hygiene and Tropical Medicine, London, UK; 2 School of Biology, University of St Andrews, St Andrews, UK

**Keywords:** eusociality, haplodiploidy, Hymenoptera, inclusive fitness, kin selection, promiscuity

## Abstract

Monogamy is associated with sibling-directed altruism in multiple animal taxa, including insects, birds and mammals. Inclusive-fitness theory readily explains this pattern by identifying high relatedness as a promoter of altruism. In keeping with this prediction, monogamy should promote the evolution of voluntary sterility in insect societies if sterile workers make for better helpers. However, a recent mathematical population-genetics analysis failed to identify a consistent effect of monogamy on voluntary worker sterility. Here, we revisit that analysis. First, we relax genetic assumptions, considering not only alleles of extreme effect—encoding either no sterility or complete sterility—but also alleles with intermediate effects on worker sterility. Second, we broaden the stability analysis—which focused on the invasibility of populations where either all workers are fully sterile or all workers are fully reproductive—to identify where intermediate pure or mixed evolutionarily stable states may occur. Third, we consider a broader range of demographically explicit ecological scenarios relevant to altruistic worker non-reproduction and to the evolution of eusociality more generally. We find that, in the absence of genetic constraints, monogamy always promotes altruistic worker sterility and may inhibit spiteful worker sterility. Our extended analysis demonstrates that an exact population-genetics approach strongly supports the prediction of inclusive-fitness theory that monogamy promotes sib-directed altruism in social insects.

## 1. Introduction

Altruism among animals is epitomized by the workers of eusocial insect societies, who sacrifice their personal reproductive success to promote their siblings’ welfare [1]. This remarkable self-abnegation—seemingly at odds with the ‘survival of the fittest’—is traditionally explained by kin selection: a gene causing workers to share provisions or defend the communal nest can spread if the workers’ sacrifice increases the survival of their siblings, who are likely to carry copies of the same gene [2–4]. Higher genetic relatedness between the altruist and her beneficiaries would therefore—all else being equal—promote selection for altruism [2]. Accordingly, monogamy is often highlighted as a key promoter of sibling altruism, because maternal promiscuity decreases relatedness between siblings, diminishing the inclusive-fitness benefits of sib-rearing [5–12]. A wealth of empirical evidence supports this view, revealing a strong association between monogamy and sib-directed altruism in insects [8,13,14], birds [15] and mammals [16].

Worker sterility in the social Hymenoptera (wasps, bees and ants) appears to be a conspicuous example of sib-directed altruism. In many hymenopteran species, female workers lay unfertilized eggs in their natal colony, which develop into males on account of their haplodiploid mode of sex determination. But in some species, workers have partly or entirely stopped making sons in order to focus their efforts on helping instead [4]. Accordingly, worker sterility may represent a trade-off between personal reproduction and sibling welfare, similarly to how the evolution of eusociality involves individuals forgoing dispersal in order to join an unmated worker caste [8–10]. As both empirical [13] and theoretical [12,17,18] studies have demonstrated that high relatedness promotes the evolution of a sterile worker caste, a standard account of inclusive-fitness theory might predict that—as with other forms of sibling altruism—monogamy should promote voluntary worker sterility.

However, this prediction has recently been challenged by Olejarz *et al.*’s [19] mathematical analysis of worker sterility in haplodiploid insect colonies, which uses an explicit population-genetics model to derive exact conditions for the invasion and stability of a worker-sterility allele. Surprisingly, this analysis could not identify a consistent effect of monogamy on the evolution of non-reproductive workers. Here, we revisit this analysis, exploring alternative assumptions concerning the genetics, evolution and ecology of worker sterility. We find that a more-comprehensive investigation of Olejarz *et al.*’s [19] exact population-genetics approach strongly supports the view that monogamy promotes altruistic worker sterility in insect societies and corroborates inclusive-fitness theory more generally.

## 2. Model and results

Olejarz *et al.* [19] investigated the spread of an allele that renders workers carrying the allele—who would otherwise produce sons through arrhenotokous parthenogenesis, substituting them for the queen’s sons—completely sterile. As the proportion *z* of sterile workers in a colony increases, the proportion *p*_*z*_ of surviving males produced by the queen rather than by workers also increases, while overall colony productivity *r*_*z*_ may increase or decrease. Reproductive females are assumed to mate *n* times before colony founding, such that varying *n* allows alternative scenarios of monogamy versus promiscuity (i.e. single versus multiple insemination) to be explored. Following these assumptions, Olejarz *et al.* [19] found that—in a seeming challenge to inclusive-fitness theory—voluntary worker sterility sometimes invades under single mating (*n*=1) only, sometimes under double mating (*n*=2) only, sometimes under both single and double mating, and sometimes under neither, suggesting no clear effect of monogamy on the invasion of sterility.

To explore the generality of this unexpected finding, we take up a suggestion by Olejarz *et al.* [19], p. 13 and extend their analysis to consider alleles with intermediate effects on worker sterility (as was done for a similar model by Olejarz *et al.* [20]). Intermediate-effect alleles may exhibit incomplete penetrance (such that each carrier has some intermediate probability of being sterile), or may encode intermediate phenotypes (such that each carrier divides her resources between colony tasks and personal reproduction); these scenarios are mathematically equivalent, but for ease of comparison with Olejarz *et al.* [19], we focus on the former interpretation. This suggested extension seems particularly apt, as the incomplete penetrance of sterility has been shown to be important for the evolution of reduced worker reproduction both in theory and in empirical practice [6,21–23]; indeed, the model of Olejarz *et al.* [19] assumes that sterility alleles are expressed only in workers, not in queens, so it is conceivable that sterility alleles may arise that are only expressed in a fraction of the workers who carry them. Accordingly, we have derived exact conditions for the invasion of a recessive or dominant sterility allele with arbitrary penetrance *v*, where *v*=1 represents full penetrance and 0<*v*<1 represents incomplete penetrance (see Methods).

Before continuing, we will clarify some assumptions and details of terminology. First, we adopt the assumption of Olejarz *et al.* [19] that worker sterility is voluntary—i.e. controlled by genes present in the worker herself. However, reduced worker reproduction could instead result from policing by other workers [21,24–26] or from manipulation by the queen [12,27,28–32]. The question of who controls worker sterility is critically important, because while monogamy ought to promote voluntary sterility [12,13,17,18], it should have no effect on maternally manipulated sterility [32,12], and is known to inhibit policing of worker reproduction by other workers [26,22].

Second, we focus on the case where worker sterility is altruistic, i.e. where workers sacrifice their personal reproduction such that the queen and any other laying workers can reproduce more. The alternative is that worker sterility involves spite [33] rather than pure altruism, such that in giving up her own reproduction, a worker reduces the fitness of the queen or of other workers. The model of Olejarz *et al.* [19] allows spiteful worker sterility to be analysed, which is a strength of their model so long as the fundamental difference between spiteful and altruistic sterility is acknowledged. We focus on non-spiteful sterility in the main text. In the Methods, we provide a mathematical definition of spiteful worker sterility and show how spiteful worker sterility may be inhibited, rather than promoted, by monogamy—an already well-established result in the inclusive fitness literature, where workers investing in suppressing other workers’ reproduction is known as worker policing [21,22,24–26].

Finally, we are focusing on the evolution of sterility among workers, and therefore we are assuming that a non-dispersing, unmated worker caste already exists. Olejarz *et al.* [19] set their results in contrast with Boomsma’s [8] ‘monogamy hypothesis’, which holds that monogamy promotes eusociality. But this contrast is potentially misleading [34,35], because the evolution of sterility among workers and the evolution of eusociality *per se*are separate things. We focus on the evolution of worker sterility as an elaboration—rather than as an inseparable feature—of eusociality, but briefly analyse the impact of monogamy on the evolution of an unmated (and sterile) worker caste at the end of the Model and results section.

### 2.1. Unconstrained allelic effects: monogamy promotes worker sterility

In this section, we analyse the invasion of voluntary worker sterility into a population with fully reproductive workers. In their analysis, Olejarz *et al.* [19] found that sterility can sometimes invade under promiscuity but not under monogamy, depending on how worker sterility affects colony productivity and the queen’s share of male production. This finding seems to contradict inclusive-fitness theory, because it apparently identifies cases where monogamy inhibits sibling-directed altruism instead of promoting it. We argue here that this conclusion is premature: sometimes because it rests on unjustified assumptions concerning the genetics of worker sterility, and sometimes because it confuses altruism with spite. In our extended invasion analysis, we allow worker-sterility alleles exhibiting incomplete penetrance or intermediate effects to arise, and we focus on altruistic worker sterility, rather than assuming that worker sterility may be spiteful. Accordingly, we find that there are no conditions under which altruistic worker sterility can invade under promiscuity and not under monogamy, and that monogamy is sometimes required for altruistic worker sterility to invade. In this sense, we show that monogamy always promotes the invasion of altruistic worker sterility relative to promiscuity.

We begin by considering the invasion of recessive worker-sterility alleles; we show that monogamy is always more favourable to the invasion of altruistic worker sterility than promiscuity (in the sense explained above), and we explain why allowing alleles of intermediate penetrance to arise overturns the result of Olejarz *et al.* [19] that double mating can be more favourable to the invasion of altruistic worker sterility than single mating. Then, we perform a similar analysis for dominant worker-sterility alleles, showing that monogamy is usually—but not always—more favourable to the invasion of altruistic worker sterility than promiscuity. Finally, we show that under the most general assumptions—namely, when we assume that worker sterility alleles could be dominant, recessive or incompletely dominant—monogamy is always more favourable to the invasion of altruistic worker sterility than promiscuity.

To facilitate comparison of our results with those of Olejarz *et al.* [19], in this section, we only consider whether—starting with a population in which no workers exhibit sterility—it is possible for a ‘sterility allele’ to invade, thereby rendering some workers sterile. Olejarz *et al.* [19] do not consider the equilibrium level of sterility that is expected to evolve in monogamous versus promiscuous populations, but focus on whether a sterility allele can invade from rarity to any non-zero frequency. We address the same question here, performing a more extensive analysis in the next section.

#### 2.1.1. Recessive worker-sterility alleles only

**Figure 1.**
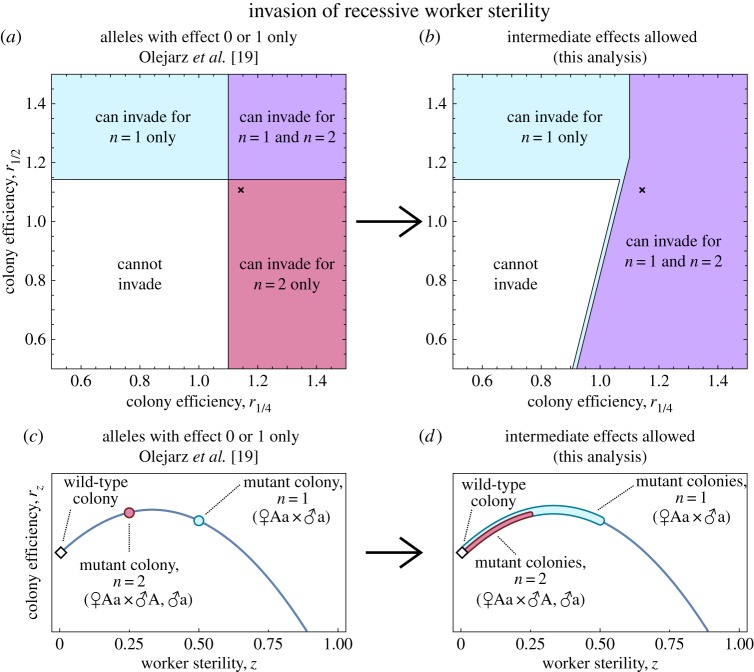
The invasion of worker sterility under recessive genetics, showing where sterility can invade under single mating (*n*=1) only, double mating (*n*=2) only, both, or neither, as a function of the colony efficiency values *r*_0_=1, *r*_1/4_ and *r*_1/2_. (*a*) If we assume that only full-sterility alleles can arise, as Olejarz *et al.* [19] did, double mating sometimes promotes the invasion of sterility relative to single mating. But (*b*) if we assume that alleles encoding any level of intermediate worker sterility may arise, double mating never promotes the invasion of sterility relative to single mating. This is because (*c*) for a rare allele encoding full sterility, mutant colonies have the phenotype $z=\frac{1}{2}$ under single mating and $z=\frac{1}{4}$ under double mating. Therefore, sterility may invade more easily under double mating if colony efficiency is relatively peaked near $z=\frac{1}{4}$. But (*d*) for a rare allele encoding intermediate sterility, mutant colonies may express any phenotype $0<z\leq \frac{1}{2}$ under single mating and $0<z\leq \frac{1}{4}$ under double mating, depending on the allele’s penetrance or effect, and so mutant phenotypes are less constrained by the population’s mating number. To facilitate comparison with fig. 3A of Olejarz *et al.* [19], we assume *p*_*z*_=0.2+0.8*z*. For *r*_*z*_, we use the unique quadratic curve passing through the points specified by *r*_0_=1, *r*_1/4_ and *r*_1/2_, but the result that single mating always promotes the invasion of recessive, non-spiteful sterility relative to double mating holds regardless of the shape of the *r*_*z*_ curve passing through these points.

When we assume that worker-sterility alleles are necessarily recessive, and require all mutant worker-sterility alleles to show full penetrance (i.e. *v*=1), our analysis exactly recovers Olejarz *et al.*’s [19] results (figure 1*a*). However, when we assume that recessive mutant worker-sterility alleles may arise with any level of penetrance (i.e. 0<*v*≤1), we find that—strikingly—monogamy always promotes the invasion of worker sterility (figure 1*b*). To be specific, we mean that if a series of worker-sterility alleles were to arise in a non-sterile population, with each allele exhibiting a randomly selected penetrance, there are no *r*_*z*_ and *p*_*z*_ curves such that at least one allele could invade under promiscuity, but no allele could invade under monogamy (provided that worker sterility is non-spiteful; see Methods). Conversely, there are an infinite number of *r*_*z*_ and *p*_*z*_ curves for which at least one recessive worker-sterility allele could invade under monogamy, but no recessive worker-sterility alleles could invade under promiscuity.

Why does allowing incomplete penetrance—or intermediate effects more generally—make such a categorical difference? The population genetics of invasion from rarity is the key. Specifically, whether a recessive sterility allele invades depends upon what happens in colonies founded by a heterozygous female who has mated with one mutant male and *n*−1 wild-type males. Other colony types featuring the mutant allele occur, but are either comparatively rare (because they require more copies of the rare mutant allele among mating partners), or exhibit exactly the same phenotype as wild-type colonies (because sterility is expressed only when both parents pass the recessive mutant allele to their daughters). Therefore, sterility can only invade if these ‘mutant’ colonies—in which a proportion *z*=*v*/2*n* of workers are sterile—succeed in spreading the sterility allele. If we only permit alleles with full penetrance (*v*=1) to arise, this allelic constraint may overpower the altruism-promoting effect of higher relatedness: for example, double mating (*n*=2) may facilitate the invasion of sterility relative to single mating (*n*=1) if colony efficiency is relatively high when $z=\frac{1}{4}$ and relatively low when $z=\frac{1}{2}$ (figure 1*c*). By contrast, if we permit alleles with incomplete penetrance (0<*v*≤1) to arise, mutant colonies may exhibit any one of a range of phenotypes, depending on *v* (namely, $0<z\leq \frac{1}{2}$ for single mating, and $0<z\leq \frac{1}{4}$ for double mating), and monogamy always promotes the invasion of worker sterility relative to promiscuity, by both maximizing sibling relatedness and allowing a wider range of phenotypes to be explored (figure 1*d*).

#### 2.1.2. Dominant worker-sterility alleles only

**Figure 2.**
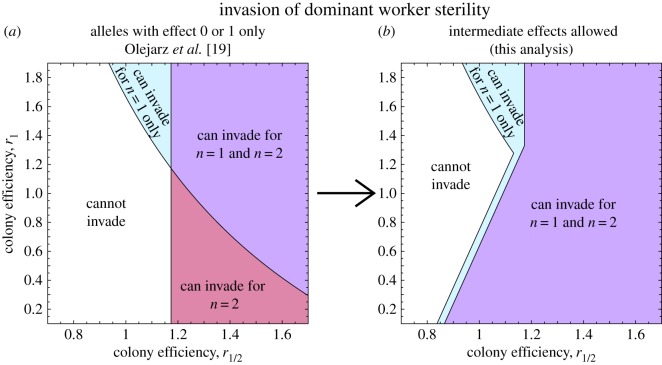
The invasion of worker sterility under dominant genetics, exploring the regions of parameter space where sterility can invade under single mating (*n*=1) only, double mating (*n*=2) only, both or neither. (*a*) If we assume that only full-sterility alleles can arise, as Olejarz *et al.* [19] did, double mating sometimes promotes the invasion of sterility relative to single mating. But (*b*) if we assume that alleles encoding intermediate worker sterility can arise, double mating does not promote the invasion of sterility relative to single mating. For comparison with fig. 8 of Olejarz *et al.* [19], we assume *p*_*z*_=0.2+0.8*z*, and for *r*_*z*_ we use the unique quadratic curve passing through the points specified by *r*_0_=1, *r*_1/2_ and *r*_1_.

If we assume that worker-sterility alleles are necessarily dominant, then there are two ‘mutant’ mating types which determine whether sterility can invade: a heterozygous mutant female mating with *n* wild-type males, and a wild-type female mating with one mutant male and *n*−1 wild-type males. These mating types produce colonies with a proportion *z*=*v*/2 and *z*=*v*/*n* of sterile workers, respectively. Hence, under single mating (*n*=1), it is the relative success of colonies with a fraction *v*/2 or *v* of sterile workers which determines whether a dominant sterility allele can invade, while under double mating (*n*=2), only the relative success of colonies with *v*/2 sterile workers determines whether a dominant sterility allele can invade. Therefore, if the relative success of colonies with a fraction *v*/2 of sterile workers is low, it is possible for single mating to disfavour the invasion of a worker-sterility allele relative to double mating. Nonetheless, for the scenario investigated by Olejarz *et al.* [19], fig. 8, we find that single mating always promotes the invasion of dominant sterility relative to double mating (figure 2).

#### 2.1.3. Any worker-sterility alleles

Above, we have considered the invasion of recessive and of dominant worker-sterility alleles as separate cases to facilitate comparison with the analysis of Olejarz *et al.* [19]. However, there is no biological reason to restrict our analysis to the cases where *either* all possible worker-sterility alleles must be recessive *or* all possible worker-sterility alleles must be dominant. If we simply make the assumption that both dominant and recessive worker-sterility alleles may arise, then—again assuming worker sterility is non-spiteful—it is not possible to construct *r*_*z*_ and *p*_*z*_ such that at least one sterility allele can invade under promiscuity, and yet no sterility allele can invade under monogamy (table 1). (The invasion of a worker-sterility allele with incomplete dominance *h*=*v* is mathematically equivalent to the invasion of a dominant worker-sterility allele with penetrance *v*, so the case of additivity or incomplete dominance does not need to be considered separately.) Hence, when arbitrary constraints on allelic variation are lifted, monogamy always promotes the invasion of worker sterility relative to promiscuity.

**Table 1.** When we assume that both recessive and dominant worker-sterility alleles may arise, and that they may exhibit incomplete penetrance, single mating (*n*=1) always promotes the invasion of non-spiteful worker sterility relative to double mating (*n*=2). For each row, 100 000 numerical experiments are performed. For each experiment, an *r*_*z*_ function is constructed using the specified procedure (see figure 3*a* and Methods for more details) and a *p*_*z*_ function is constructed such that, by forfeiting male egg production, a worker either increases or decreases other workers’ reproductive success (in the latter case, worker sterility is spiteful; see Methods). Then we see whether it is possible for any worker-sterility allele—whether dominant or recessive, and of any non-zero penetrance—to invade under single mating and under double mating. Here, we test alleles with penetrance *v* in the set {0.1,0.2,0.3,…,1} and report the number of cases in which at least one sterility allele can invade. Equivalent results hold if we only test alleles with penetrance 0.5 or 1, illustrating that the amount of available genetic variation does not need to be extensive for monogamy to promote the invasion of worker sterility relative to promiscuity. Note that the spiteful versus non-spiteful sterility distinction here relates only to the *p*_*z*_ function (i.e. worker-directed spite; see Methods).

	non-spiteful worker sterility			
	number of cases in which a sterility allele can invade…			
	_______________________________________________________________________			
procedure for generating *r*_*z*_	for *n*=1 only	for *n*=2 only	for both *n*=1 and *n*=2	for neither *n*=1 nor *n*=2
(i) random noise	10 304	0	55 841	33 855
(ii) plateau	6587	0	45 679	47 734
(iii) random steps	7401	0	49 079	43 520
(iv) increasing steps	7593	0	90 299	2108
(v) linear	4142	0	41 183	54 675
	spiteful worker sterility			
	number of cases in which a sterility allele can invade…			
	_______________________________________________________________________			
procedure for generating *r*_*z*_	for *n*=1 only	for *n*=2 only	for both *n*=1 and *n*=2	for neither *n*=1 nor *n*=2
(i) random noise	3738	1840	64 996	29 426
(ii) plateau	1474	1528	51 717	45 281
(iii) random steps	3151	1223	55 007	40 619
(iv) increasing steps	1542	0	98 189	269
(v) linear	896	0	46 105	52 999

### 2.2. Beyond invasion: monogamy promotes worker sterility

**Figure 3.**
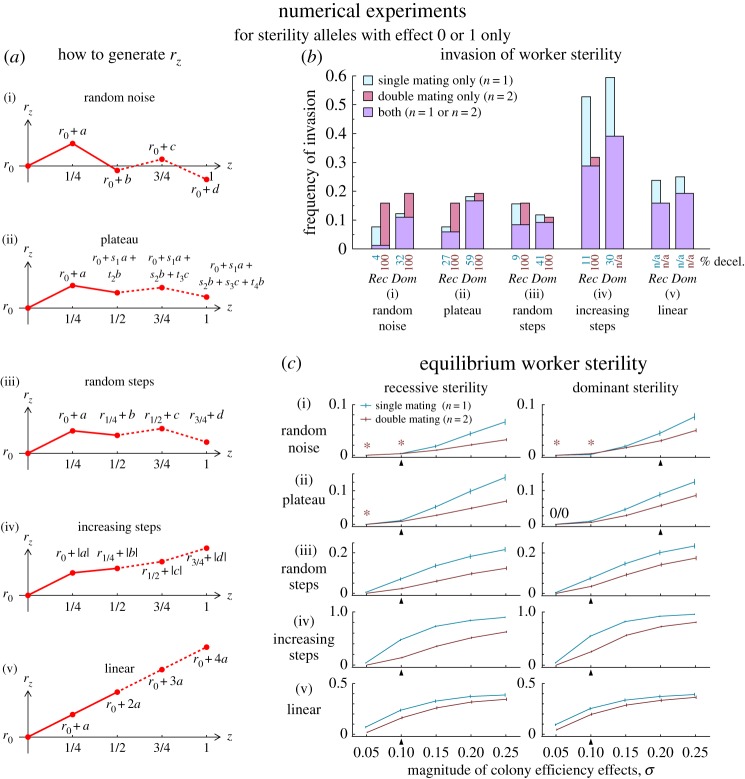
Here, we compare the evolution of worker sterility under single (*n*=1) versus double mating (*n*=2) by revisiting the numerical experiments of Olejarz *et al.* [19]. (*a*) There are many possible ways to construct the colony efficiency function *r*_*z*_ based on picking random numbers from a normal distribution. Five alternatives are shown here, including the two procedures used by Olejarz *et al.* (‘Random noise’, their Procedure 1, and ‘Plateau’, their Procedure 2; see Methods). For testing whether sterility invades, only two points are needed (solid lines), but this can be extended to four points (dashed lines) for measuring sterility at equilibrium. (*b*) We record the frequency of invasion of a full-sterility allele under single versus double mating, running 10 million experiments for each scenario. Percentages beneath the bar chart show that an initially decelerating *r*_*z*_ is required for sterility to invade under double mating only (see Methods). (*c*) We record the average worker sterility at equilibrium over 5000 experiments for each scenario. Except when *r*_*z*_ is constructed using the ‘random noise’ or ‘plateau’ procedure for a small magnitude of efficiency effects (asterisks), single mating tends to promote average worker sterility at equilibrium relative to double mating (the 0/0 denotes no worker sterility under either single or double mating). This can happen even if sterility is more likely to invade under double mating (for example, compare results of procedures (i)–(iii) in (*b*) versus (*c*)). Arrowheads beneath the *x*-axis show where parameters coincide with those used in (*b*). The ‘magnitude of colony efficiency effects’ is the standard deviation of normally distributed variates used for constructing *r*_*z*_. For (*b*) and (*c*), we assume *p*_*z*_=0.2+0.8*z*. See Methods for details.

We have shown that, by relaxing the strong genetic constraints imposed by the analysis of Olejarz *et al.* [19], monogamy always promotes the invasion of non-spiteful worker sterility relative to promiscuity. But to only consider whether sterility alleles can invade may be misleading, for two reasons. First, that a sterility allele spreads from rarity says little about its equilibrium frequency, which may be a more-relevant measure of monogamy’s impact upon worker altruism than mere invasion. Indeed, although as Olejarz *et al.* have shown promiscuity sometimes promotes sterility’s invasion *per se* under full penetrance, we find that monogamy typically increases the equilibrium level of sterility under the same conditions. Interestingly, we find that the ‘numerical experiments’ of Olejarz *et al.*, which identified more cases in which only double mating promoted the invasion of sterility than cases in which only single mating promoted the invasion of sterility, are highly sensitive to the method used to construct the colony productivity function *r*_*z*_ (figure 3).

**Figure 4.**
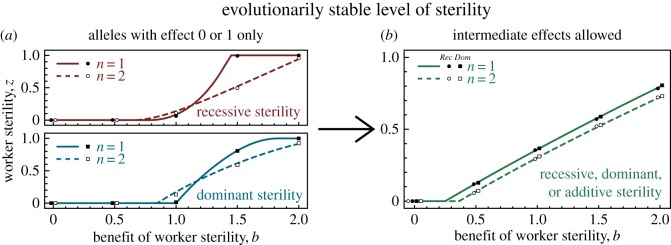
The evolutionarily stable level of sterility under single (*n*=1) versus double mating (*n*=2), for (*a*) constrained allelic variation, with recessive (i) versus dominant (ii) sterility and (*b*) unconstrained allelic variation, regardless of whether sterility is recessive, dominant or additive. (*a*) When allelic variation is constrained, double mating (dashed lines) can sometimes promote sterility relative to single mating (solid lines). But (*b*) when allelic variation is unconstrained, single mating always promotes sterility. Overlaid markers show results of a stochastic individual-based model (see Methods), matching well with the predicted evolutionarily stable levels of worker sterility. To illustrate a scenario where constraints on heritable variation may lead to promiscuity promoting worker sterility relative to monogamy, we use the colony efficiency function *r*_*z*_=1+*bz*−*z*^2^, with a ‘benefit of worker sterility’ term *bz* and a ‘decelerating’ term −*z*^2^. For the proportion of male eggs laid by the queen, we again use *p*_*z*_=0.2+0.8*z*.

Second, if we do allow intermediate-effect alleles, then considering only whether a single invasion occurs is inadequate, because long-term evolution is likely to involve multiple successive invasions (cf. [36]). How can we predict the outcome without knowing in advance which alleles may arise, and when? The solution is that, over the long term, populations exposed to sufficient genetic variation will converge on an evolutionarily stable strategy (ESS; [37])—a level of sterility that cannot be invaded by an allele encoding any other level of sterility. To identify a candidate ESS for sterility, we further extend Olejarz *et al.*’s [19] population-genetics analysis to derive an exact condition for the invasion of an allele encoding a small increase to average sterility, *z*:

2.1 $$-\frac{1}{1-z}(1-p_{z})(3n-2)+\frac{r_{z}^{\prime }}{r_{z}}(4+3n(1+p_{z}))-p_{z}^{\prime }(2-n)>0,$$

where *r*′_*z*_ and *p*′_*z*_ are the slopes of the *r*_*z*_ and *p*_*z*_ functions at *z*, respectively. Remarkably, this exact condition holds for both recessive and dominant genetics. Using this condition and a global stability analysis, we find that the ESS for sterility is always at its highest under single mating (figure 4; see Methods).

Intuition for this exact population-genetics result may be obtained by recasting condition (2.1) in terms of inclusive fitness [2]. Accordingly, natural selection favours an increase to average sterility, *z*, when

2.2 $$\underset{\mathrm{sacrifice}\;\mathrm{effect}}{\underbrace{-\frac{1-p_{z}}{1-z}R_{\mathrm{son}}}}+\underset{\mathrm{efficiency}\;\mathrm{effect}}{\underbrace{\frac{r_{z}^{\prime }}{r_{z}}(R_{\mathrm{sis}}+p_{z}R_{\mathrm{bro}}+(1-p_{z})R_{\mathrm{neph}})}}+\underset{\mathrm{male}\;\mathrm{production}\;\mathrm{effect}}{\underbrace{p_{z}^{\prime }R_{\mathrm{bro}}+\left(\frac{1-p_{z}}{1-z}-p_{z}^{\prime }\right)R_{\mathrm{neph}}}}>0,$$

where $R_{\mathrm{son}}=\frac{1}{2}$, *R*_neph_=(2+*n*)/8*n*, *R*_sis_=(1+*p*_*z*_)((2+*n*)/8*n*) and $R_{\mathrm{bro}}=\frac{1}{4}$ are the life-for-life relatedness of a worker to her son, her nephew (a random worker’s son), her reproductive sister and her brother, respectively [5]. Note that promiscuity decreases worker relatedness to sisters and nephews, but not to sons or brothers. Hence, when worker sterility is non-spiteful, monogamy always increases selection for sterility.

The left-hand side of condition (2.2) can be interpreted as the inclusive-fitness effect experienced by a focal worker who stops laying male eggs. The ‘sacrifice effect’ captures the direct cost of her sterility, in that she forfeits her relative share (1−*p*_*z*_)/(1−*z*) of all worker-laid males. The ‘efficiency effect’ captures her impact on colony efficiency, which increases by a relative amount *r*′_*z*_/*r*_*z*_, augmenting the production of her sisters and of colony-produced males, a proportion *p*_*z*_ of whom are her brothers and a proportion 1−*p*_*z*_ of whom are her nephews. And the ‘male production effect’ captures her impact on the proportion of male eggs produced by the queen versus workers: her relative gain of brothers is *p*′_*z*_, while her relative gain or loss of nephews exactly balances her forfeited sons and gained brothers.

Condition (2.2) clarifies the impact of monogamy upon worker sterility: by increasing a worker’s relatedness to her nephews and sisters, monogamy increases her inclusive-fitness benefit of promoting colony efficiency, and by increasing a worker’s relatedness to her nephews, it increases her inclusive-fitness benefit of augmenting her fellow workers’ production of sons. Hence, overall, monogamy promotes non-spiteful worker sterility. Note that if sterility either reduces colony efficiency (*r*′_*z*_<0) or reduces the reproduction of other workers ((1−*p*_*z*_)/(1−*z*)−*p*′_*z*_<0), then worker sterility is spiteful and may be relatively promoted by promiscuity (see Methods). Condition (2.2) also clarifies how Olejarz *et al.*’s [19] model differs from Boomsma’s [8–10] model for the evolution of eusociality: in Boomsma’s model, females trade away offspring for siblings as dispersers evolve into a non-totipotent worker caste, while in Olejarz *et al.*’s model, an existing non-totipotent worker caste trades away sons for brothers and nephews. Conditions (2.1) and (2.2) are exactly equivalent, are valid for recessive, dominant or additive genetics, and can be obtained using standard kin-selection methodology (see Methods).

**Figure 5.**
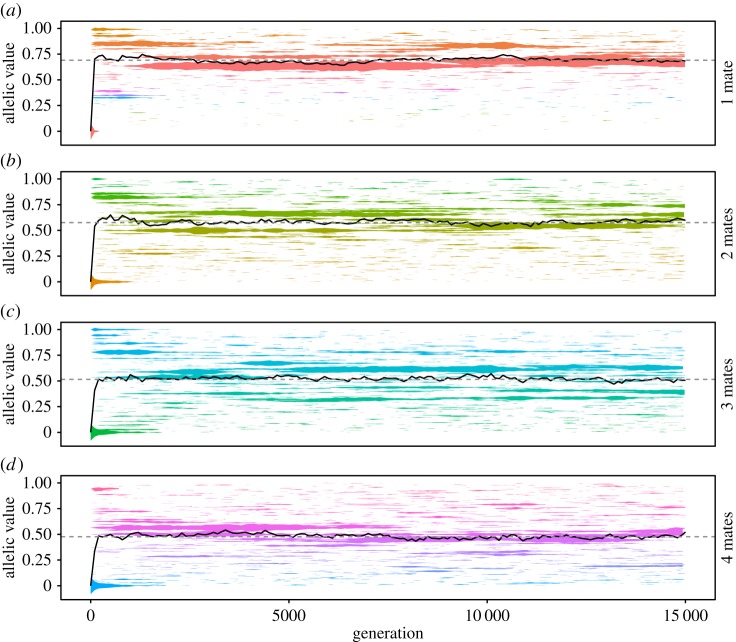
Predicted evolutionarily stable states for worker sterility are valid even assuming a ‘saltationist’ model of genetic mutation in which newly arising alleles take a random penetrance uniformly distributed between 0 and 1. Separate plots show increasing levels of promiscuity, from *n*=1 mate (*a*) to *n*=4 mates (*d*). Within a plot, the predicted ESS for sterility, z*, is shown as a dashed line; the average worker sterility in a given generation is shown as a solid line; and coloured ribbons show the alleles present in the population, with the width of each ribbon giving the allele’s relative frequency and the vertical position of the ribbon giving the allele’s penetrance. We assume that sterility is controlled by a single locus at which allelic effects are averaged together, but results are equivalent for fully dominant or fully recessive alleles, or when we assume that sterility is controlled by multiple loci, each with different magnitudes of allelic effect. Alleles which persist for fewer than 100 generations are not shown in this figure. We assume that *r*_*z*_=1+*z*−0.5*z*^2^ and *p*_*z*_=0.2+0.8*z*, which yields z*=0.690 when *n*=1; z*=0.576 when *n*=2; z*=0.515 when *n*=3; and z*=0.476 when *n*=4.

Conditions (2.1) and (2.2) can be derived using either the simplifying assumption that genetic variation for worker sterility is at a single locus and that new allelic variants arise via mutations of vanishingly small effect (see appendix A), or using the more general assumption that worker sterility is a quantitative trait (see appendix B). However, it is important to note that their utility in predicting an equilibrium level of worker sterility extends beyond these cases. Using an individual-based model (see Methods) to analyse a population in which mutant sterility alleles of any penetrance 0≤*v*≤1 may arise—not just those exhibiting incremental differences in penetrance—we find that the ESS predicted by conditions (2.1) and (2.2) is still reached and that monogamy still promotes the evolution of worker sterility relative to promiscuity (figure 5).

### 2.3. Alternative ecological scenarios: monogamy promotes worker sterility

**Figure 6.**
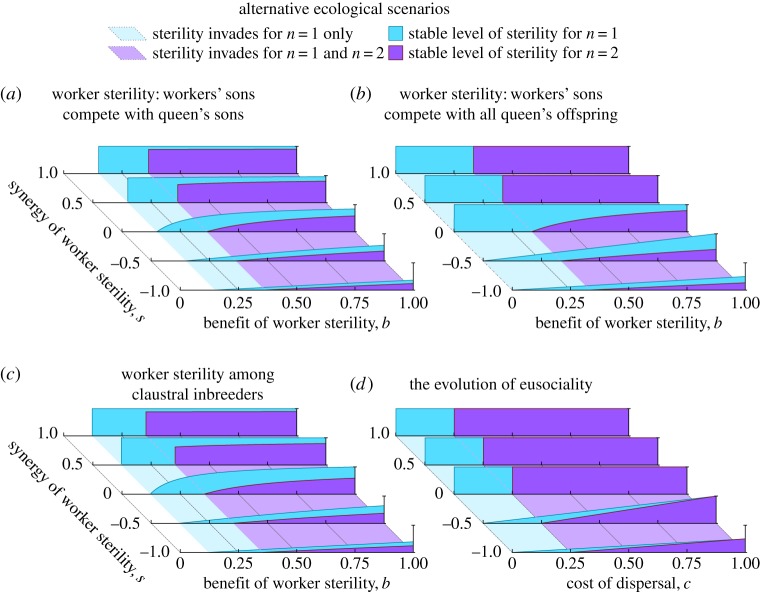
The evolution of worker sterility under alternative ecological scenarios. Here, we determine the stable level of worker sterility under four demographically explicit models of worker sterility; see Methods for full details. (*a*) One possible assumption is that worker-laid males only compete with the queen’s sons (cf. [19]). In this case, monogamy promotes worker sterility relative to promiscuity. (*b*) It is also possible to assume that worker-laid males compete with the queen’s offspring of both sexes, and not just with the queen’s sons. In this case, monogamy promotes worker sterility relative to promiscuity. (*c*) In the gall-forming thrips, the foundress produces an initial brood of female and male soldiers, who may produce part of the next brood by inbreeding among themselves [39]. Female soldiers can sacrifice part of their reproductive potential to invest more in defending their nestmates. In this case, monogamy promotes worker sterility relative to promiscuity. (*d*) A possible model for the evolution of eusociality involves dispersing, fully reproductive females evolving into sterile workers, who stay in the nest to help, producing no offspring [8–10]. In this case, monogamy promotes worker sterility relative to promiscuity. We show results for *k*=4 in (*a*) and *k*=2 in (*b*) and (*c*) (see Methods for details).

**Figure 7.**
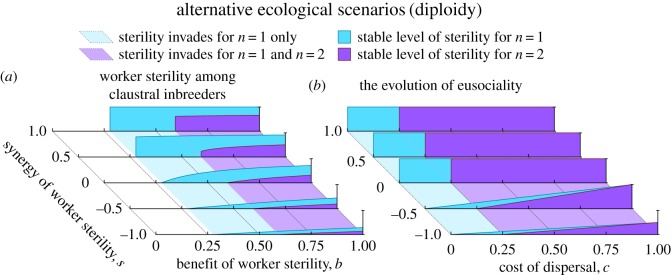
The evolution of worker sterility under alternative ecological scenarios, for diploidy. Here, we determine the stable level of worker sterility under two demographically explicit models of worker sterility; see Methods for full details. (*a*) For claustral inbreeders under diploidy, monogamy promotes worker sterility relative to promiscuity; we show results for *k*=4 here. (*b*) For the evolution of eusociality via non-dispersing female workers under diploidy, monogamy promotes worker sterility relative to promiscuity. Note that while figures 6*c* and 7*a* are qualitatively similar but quantitatively different, figures 6*d* and 7*b* are identical, highlighting the varying impact of haplodiploidy versus diploidy within alternative ecological scenarios.

Finally, we consider some alternative scenarios for the evolution of worker non-reproduction, using a demographically explicit model of queen-worker competition over egg-laying (see Methods). This yields a functional form for *p*_*z*_ which explicitly accounts for the relative egg-production capabilities of workers relative to the queen, which we substitute for the more hypothesis-free linear forms of the *p*_*z*_ function analysed by Olejarz *et al.* [19]. We then use standard neighbour-modulated-fitness methodology [38] to consider four alternative scenarios. First, we consider the original scenario of Olejarz *et al.* [19], in which workers’ sons compete only with the queen’s sons. Second, we consider a scenario in which workers’ sons compete equally with the queen’s sons and daughters, which requires analysis of sex ratio evolution because the queen is selected to adjust her sex allocation in response to workers’ sons potentially replacing her daughters. Third, we consider the evolution of soldier sterility in claustral inbreeders, such as the gall-forming thrips [39]. Fourth, we consider the evolution of a sterile worker caste via female non-dispersal, i.e. a possible scenario for the evolution of eusociality [8–10]. In all four cases, we find that monogamy always promotes non-spiteful worker sterility relative to promiscuity. Strikingly, these more-realistic scenarios identify large parameter ranges over which monogamy is critical for the evolution of worker sterility or of a worker caste (figure 6; see Methods). This conclusion also holds if we alternatively consider a diploid mode of inheritance, as exhibited by termites (figure 7; see Methods).

## 3. Discussion

In seeming contrast with the predictions of inclusive-fitness theory, Olejarz *et al.*’s [19] exact population-genetics analysis could not identify a consistent effect of monogamy on the evolution of voluntary worker sterility. This surprising result, if robust, would have not only overturned a considerable theoretical consensus, but would also have left a number of empirically described patterns bereft of a predictive, explanatory framework. Happily, we have shown that by relaxing constraints on genetic variation (figures 1 and 2 and table 1), considering the consequences of invasion rather than just its occurrence (figure 3), describing long-term evolutionarily stable states (figures 4 and 5), and exploring a wide range of ecological scenarios (figures 6 and 7), a clear sterility-promoting effect of monogamy consistently emerges. Moreover, we have shown that the long-term evolutionary outcome is readily described, conceptualized and explained by standard inclusive-fitness theory. In sum, a more comprehensive analysis based on Olejarz *et al.*’s [19] exact population-genetics approach supports inclusive-fitness theory and its prediction that monogamy promotes the evolution of altruistic worker sterility.

We have found that a distinction needs to be made between non-spiteful and spiteful worker sterility. Worker sterility may be spiteful if it either decreases colony productivity (i.e. if *r*′_*z*_<0) or if, by giving up her own reproduction, a worker reduces the reproductive fitness of other workers (i.e. if *p*′_*z*_>(1−*p*_*z*_)/(1−*z*); see Methods). When worker sterility is spiteful, monogamy may inhibit worker sterility relative to promiscuity. However, this is not because inclusive-fitness predictions for the evolution of worker sterility are wrong: on the contrary, it is a straightforward consequence of condition (2.2), an exact population-genetics result that was derived without reference to inclusive fitness, but which has a clear and intuitive interpretation in terms of a worker’s inclusive fitness. This is exactly analogous to how kin-selection methodology makes diametrically opposite predictions as to patterns of social sterility in polyembryonic parasitoid wasps depending on whether the soldiers have a family-benefit or within-family-conflict function [40,41]. It is generally understood that a worker allocating resources to egg-laying will be less able to allocate resources to colony tasks. Moreover, under the simplest assumptions, a worker abstaining from male production should, in doing so, increase the relative contribution to male production of both other workers and of the queen, which would yield a non-spiteful *p*_*z*_ function (see Methods) and would lead to monogamy promoting worker sterility. A potential example of worker spite is proposed by Olejarz *et al.* who suggest that—in the context of the queen policing worker reproduction—if ‘too many workers reproduce, then the queen could be overwhelmed, and her effect on removing worker-laid eggs is diminished’ [19], p. 6. This could indeed yield a spiteful *p*_*z*_ function if, for example, the queen were so ‘overwhelmed’ by the production of an additional worker egg that she lost track of more than one elsewhere. This is not impossible, but it does seem unlikely to be generally true, and in the absence of a concrete model or empirical support for this scenario, the assertion that spiteful worker sterility is an ‘equally plausible scenario’ [19] is difficult to accept.

Crucially, we did not derive our analysis by assuming beforehand that the evolution of worker sterility is determined by a specific condition of the form *rb*>*c* (i.e. a Hamilton’s [2] rule). Instead, we began with an explicit population-genetics model which contains no ‘built-in’ assumptions about inclusive-fitness effects. Our findings differ from those of Olejarz *et al.* [19] not because we have interpreted them using inclusive-fitness theory, but fundamentally because we have relaxed the genetic assumptions made by Olejarz *et al.* and focused on the long-term outcome of evolution rather than on the success or failure of a single invasion by a worker-sterility allele of specific effect. We then presented the results of this explicit population-genetics analysis (condition (2.1)) using an inclusive-fitness interpretation (condition (2.2)) because this form is more intuitive. This underlines that the role of inclusive-fitness theory is not usually to provide the starting point for a formal mathematical analysis, but rather to provide synthesis of—and facilitate generalization beyond—the results obtained by a diversity of different analyses undertaken using a diversity of different methodologies [42].

Although our analysis demonstrates that monogamy typically promotes worker sterility even when strong genetic constraints are assumed (figure 3*c*), we focus on the result that monogamy always promotes non-spiteful worker sterility in the absence of such genetic constraints (table 1 and figures 4–7). Formally, this analysis makes the assumption of ‘weak selection’, i.e. that allelic variation is small in magnitude so that the effect of large fitness differences between genotypes can be ignored. Does this mean that we are replacing one set of unrealistic genetic assumptions (full penetrance only) with another (weak selection)? No, because weak-selection results represent the limiting case of long-term evolution under a variety of different assumptions. Indeed, our main results are robust under a variety of evolutionary scenarios. First, they can be derived using an explicit population-genetics analysis that assumes that worker sterility is controlled by infinitesimal variation appearing at one locus at a time and that worker-sterility alleles are either dominant or recessive (appendix A). Second, they can also be derived using standard kin-selection methodology [38] which assumes additive, heritable genetic variation potentially at many loci (appendix B). Finally, we have shown that these ESS predictions are reached when we assume that allelic variation may arise at one or at many loci and that mutations typically have large effects on phenotype in a finite population subject to stochastic effects (figure 5).

**Figure 8.**
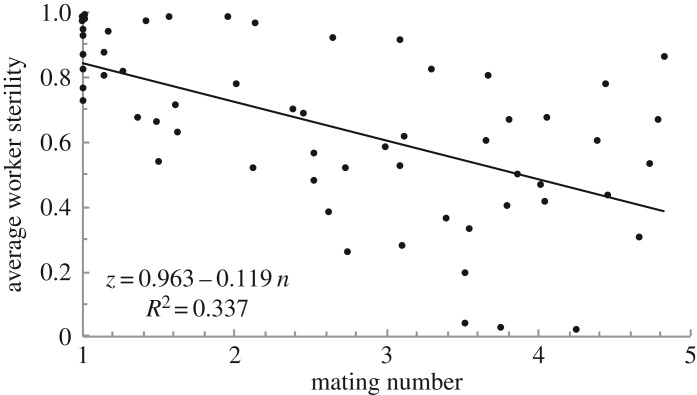
Results of a hypothetical field experiment measuring voluntary worker sterility—that is, sterility in the absence of policing [26,22] or maternal manipulation—in 60 species varying in mating number using a stochastic individual-based model (see Methods). Ten of the species have single mating (*n*=1), while 50 of the species have a mating number *n* of between 1 and 5. For each species, the colony productivity function *r*_*z*_ is a quadratic function with coefficients chosen randomly such that full worker sterility gives a 50–150% productivity increase and is equally likely to be concave or convex, and the egg production function is of the form *p*_*z*_=1/(1+*k*(1−*z*)), with *k* randomly chosen between 1 and 5. The trend is noisy, because different species face different ecological trade-offs in worker sterility. Nonetheless, a clear pattern—that monogamy is associated with higher worker sterility—emerges.

The approach of Olejarz *et al.* [19] gives exact results for the invasion of worker sterility, but under extraordinary genetic constraints, namely that sterility is determined by a single locus with either recessive or dominant alleles of full penetrance. Olejarz *et al.* point out that, under these conditions, the mating number and a few points from the *r*_*z*_ and *p*_*z*_ curves are sufficient to predict whether sterility will invade. However, we rarely have this much information about any particular population of interest, let alone for all populations for which we would intend such theory to apply. It is much more likely that we will be presented with a pattern in the natural world—e.g. that voluntary sterility tends to be more common in species with monogamous mating (figure 8)—which may well be noisy. The goal of evolutionary analysis should be, first and foremost, to provide an intuitive explanation for these broad patterns, rather than trying to provide exact but difficult-to-interpret results for an idealized scenario that will never be encountered in the real world (cf. [43]). Needless to say, ecological factors—i.e. the costs and benefits of worker sterility—play a crucial role. But relatedness is also important, and we have found that monogamy promotes altruistic worker sterility across a broad range of scenarios.

## 4. Methods

### 4.1. Spiteful worker sterility and policing

In the model of Olejarz *et al.* [19], worker spite may occur via two routes—one operating through colony efficiency, *r*_*z*_, and one operating through the queen’s production of males, *p*_*z*_. The first case occurs when an increase in average worker sterility decreases colony efficiency (i.e. when *r*′_*z*_<0)—for example, if the sterility allele has a pleiotropic effect on worker condition which results in less-efficient work. In such a case, monogamy will inhibit the evolution of worker sterility relative to promiscuity, because promiscuity decreases relatedness between relatives, thereby lessening the harmful impact of sterility upon a worker’s inclusive fitness via colony efficiency.

The second case occurs when an increase in a focal worker’s sterility harms the reproductive success of other workers. In the main text, we assume that when a worker becomes sterile, her forfeited sons are replaced partly by the queen’s sons and partly by her sisters’ sons, such that by forfeiting sons she gains both nephews and brothers, or at least does not lose nephews. But if, due to the shape of the *p*_*z*_ function, the queen gains a larger proportion of sons than the worker forfeits (that is, when *p*′_*z*_>((1−*p*_*z*_)/(1−*z*))), this ‘outsized gain’ by the queen must be balanced by *decreased* male production by other workers, such that, by becoming sterile, the focal worker loses nephews overall. If the focal worker loses nephews by becoming sterile (i.e. when (1−*p*_*z*_)/(1−*z*)−*p*′_*z*_<0; see condition (2.2)), then promiscuity, by decreasing the worker’s relatedness to nephews, may promote this spiteful form of worker sterility relative to monogamy, unless this relative cost of sterility is countered by a colony efficiency benefit of sterility, which would be largest in magnitude under monogamy.

This second form of spiteful worker sterility is connected with worker policing [24,25]. Specifically, both worker policing and this form of worker spite involve workers investing in reducing the reproduction of other workers in order to increase colony productivity. Standard inclusive-fitness theory [21,24,25] and empirical evidence [22,26] have emphasized that promiscuity promotes worker policing, so the result that this form of worker spite may be promoted by promiscuity is not at all surprising.

For non-incremental increases in sterility, the condition for spiteful worker sterility becomes (*p*_*v*_−*p*_*u*_)/(*v*−*u*)>(1−*p*_*u*_)/(1−*u*), where *u* is the level of worker sterility in the monomorphic population before the mutant allele is introduced, and *v* is the level of worker sterility encoded by the mutant allele.

### 4.2. Explicit population-genetics analysis

In appendix A, we extend the methods of Olejarz *et al.* [19] to consider the invasion of an allele with an arbitrary effect on worker sterility; the results of this analysis are presented here. We find that a recessive allele encoding worker sterility *v* can invade a population monomorphic for sterility *u* when

4.1 $$\frac{r_{((2n-1)u+v)/2n}}{r_{u}}>\frac{2(2n(1-u)+u-v)(2+n(1+p_{u}))}{\left(\begin{array}{l} n(8+4n(1-u)-3u-5v)+2(u-v)+(2+n)(2n(1-u)+u-v)p_{u}\\ \;-\;2n(2-u-v-n(1-u))p_{((2n-1)u+v)/2n} \end{array}\right)}.$$

Similarly, we find that a dominant allele encoding worker sterility *v* can invade a population monomorphic for sterility *u* when

4.2 $$\frac{r_{(u+v)/2}}{r_{u}}\left(\begin{array}{l} 1+\left(\frac{(1-u)p_{(u+v)/2}}{2-u-v}+\frac{\left(1-v)((2-n)(u-v)+n(2-u-v)p_{((n-1)u+v)/n}\right)}{2(n(1-u)+u-v)(2-u-v)}\right)\\ \;\frac{r_{((n-1)u+v)/n}}{r_{u}}+\frac{n(1-v)(1-p_{((n-1)u+v)/n})}{n(1-u)+u-v}\;\frac{r_{((n-1)u+v)/n}}{r_{(u+v)/2}} \end{array}\right)>2.$$

Note that conditions (4.1) and (4.2) give both the invasion and stability of a given level of sterility: that is, if a sterility allele with effect *v* can invade a population monomorphic for sterility *u*, then this is the same as saying that a population monomorphic for sterility *u* is not stable to invasion by a sterility allele with effect *v*. For example, substituting *n*=1, *u*=0, *v*=1 into condition (4.1) yields the condition for the invasion of a recessive sterility allele under single mating from Olejarz *et al.* [19], their condition 1, while substituting *n*=1, *u*=1, *v*=0 into condition (4.2) yields the condition for the stability of a recessive sterility allele under single mating from Olejarz *et al.* [19], their condition 3.

To find when natural selection will favour a small increase in sterility *δz*, we make the substitution *v*=*u*+*δz* into conditions (4.1) and (4.2) above. Then, by linearizing *r*_*z*_ and *p*_*z*_ around the point *z*=*u*, we can recast these conditions in terms of the value and slope of *r*_*z*_ and *p*_*z*_ at this point. More specifically, for a recessive sterility allele, substituting *v*=*u*+*δz* into condition (4.1) yields

$$\frac{r_{u+\delta z/2n}}{r_{u}}>\frac{2(2n(1-u)-\delta z)(2+n(1+p_{u}))}{\left(\begin{array}{l} 4n(2+n)(1-u)-(2+5n)\delta z+(2+n)(2n(1-u)-\delta z)p_{u}\\ \;-\;2n(2-n-(2-n)u-\delta z)p_{u+\delta z/2n} \end{array}\right)}.$$

Linearizing *r*_*z*_ and *p*_*z*_ around *z*=*u*, we replace *r*_*u*+*δz*/2*n*_ with *r*+(*δz*/2*n*)*r*′, where *r*=*r*_*u*_ and *r*′=*dr*/*dz*|_*z*=*u*_. Similarly, we replace *p*_*u*+*δz*/2*n*_ with *p*+(*δz*/2*n*)*p*′, where *p*=*p*_*u*_ and *p*′=*dp*/*dz*|_*z*=*u*_. This yields

$$\frac{r+(\delta z/2n)r^{\prime }}{r}>\frac{2(2n(1-u)-\delta z)(2+n(1+p))}{\left(\begin{array}{l} 4n(2+n)(1-u)-(2+5n)\delta z+(2+n)(2n(1-u)-\delta z)p\\ \;-\;2n(2-n-(2-n)u-\delta z)(p+(\delta z/2n)p^{\prime }) \end{array}\right)}.$$

Eliminating the fractions on both sides, discarding terms of order *δz*^2^ or higher, substituting *z* for *u* and simplifying yields

$$-\frac{1}{1-z}(1-p_{z})(3n-2)+\frac{r_{z}^{\prime }}{r_{z}}(4+3n(1+p_{z}))-p_{z}^{\prime }(2-n)>0,$$

which is condition (2.1) of the main text.

Similarly, for a dominant sterility allele, substituting *v*=*u*+*δz* into condition (4.2) yields

$$\frac{r_{u+\delta z/2}}{r_{u}}\left(\begin{array}{l} 1+\left(\frac{(1-u)p_{u+\delta z/2}}{2-2u-\delta z}+\frac{\left(1-u-\delta z)(n(2-2u-\delta z)p_{u+\delta z/n}-(2-n)\delta z\right)}{2(n(1-u)-\delta z)(2-2u-\delta z)}\right)\frac{r_{u+\delta z/n}}{r_{u}}\\ \;+\frac{n(1-u-\delta z)(1-p_{u+\delta z/n})}{n(1-u)-\delta z}\frac{r_{u+\delta z/n}}{r_{u+\delta z/2}} \end{array}\right)>2.$$

By linearizing *r*_*z*_ and *p*_*z*_ around *z*=*u* as above, we obtain

$$\frac{r+(\delta z/2)r^{\prime }}{r}\left(\begin{array}{l} 1+\left(\frac{\left(1-u)(p+(\delta z/2)p^{\prime }\right)}{2-2u-\delta z}+\frac{\left(1-u-\delta z)(n(2-2u-\delta z)(p+(\delta z/n)p^{\prime })-(2-n)\delta z\right)}{2(n(1-u)-\delta z)(2-2u-\delta z)}\right)\\ \;\frac{r+(\delta z/n)r^{\prime }}{r}+\frac{n(1-u-\delta z)(1-p-(\delta z/n)p^{\prime })}{n(1-u)-\delta z}\frac{r+(\delta z/n)r^{\prime }}{r+(\delta z/2)r^{\prime }} \end{array}\right)>2.$$

Expanding all terms, discarding terms of order *δz*^2^ or higher, substituting *z* for *u* and simplifying yields

$$-\frac{1}{1-z}(1-p_{z})(3n-2)+\frac{r_{z}^{\prime }}{r_{z}}(4+3n(1+p_{z}))-p_{z}^{\prime }(2-n)>0,$$

which, again, is condition (2.1) of the main text.

### 4.3. Numerical experiments

Olejarz *et al.* [19] performed numerical experiments to see whether sterility was more likely to invade under single mating or double mating. To do so, they constructed randomly generated *r*_*z*_ functions according to one of two procedures. Here, we add to these procedures, bringing the number of possible methods for constructing the *r*_*z*_ function to five (figure 3*a*). Each involves drawing four random variates—here, notated as *a*, *b*, *c* and *d*—from a normal distribution with mean 0 and standard deviation *σ*. In all cases, we assume *r*_0_=1, and use the random variates to generate *r*_1/4_, *r*_1/2_, *r*_3/4_ and *r*_1_, which suffice to numerically integrate the evolutionary dynamics of worker sterility using the system of ODEs described by Olejarz *et al.* [19]. We restrict our attention here to the invasion of an allele encoding full sterility in its carriers, under either recessive or dominant genetics.

The first procedure, ‘random noise’, is equivalent to Procedure 1 in Olejarz *et al.* [19]. Here, we set *r*_1/4_=*r*_0_+*a*, *r*_1/2_=*r*_0_+*b*, *r*_3/4_=*r*_0_+*c* and *r*_1_=*r*_0_+*d*. Note that the four values are completely uncorrelated with each other; sequential values of *r*_*z*_ are independent from previous values, which is why we have named this procedure ‘random noise’. This procedure might generate plausible *r*_*z*_ functions for a population where every colony-level increase in worker sterility were to completely erase the effect of any previous increase in worker sterility, replacing it with a new, random effect. That is, it is not particularly plausible.

The second procedure, ‘plateau’, is equivalent to Procedure 2 in Olejarz *et al.* [19]. Here, the values *r*_1/4_, *r*_1/2_, *r*_3/4_ and *r*_1_ are drawn from a correlated multivariate normal distribution. This can be simulated by transforming four uncorrelated normal variates; one way of doing this is by using the matrix

$$\left[\begin{array}{cccc} 1 & \rho & \rho & \rho \\ \rho & 1 & \rho & \rho \\ \rho & \rho & 1 & \rho \\ \rho & \rho & \rho & 1 \end{array}\right],$$

where *ρ* is the desired correlation between each variate. By multiplying the vector of uncorrelated variates by the Cholesky decomposition of this matrix, one obtains four correlated variates

$$\begin{array}{rl} a^{\prime } & \;=a,\\ b^{\prime } & \;=a\rho +b\sqrt{1-\rho^{2}},\\ c^{\prime } & \;=a\rho +b\frac{\rho \sqrt{1-\rho }}{\sqrt{1+\rho }}+c\sqrt{3-2\rho -\frac{2}{1+\rho }}\\ \mathrm{and}\;d^{\prime } & \;=a\rho +b\frac{\rho \sqrt{1-\rho }}{\sqrt{1+\rho }}+c\frac{\rho \sqrt{3-2\rho -2/(1+\rho )}}{1+2\rho }+d\sqrt{\frac{1+\rho (2-3\rho )}{1+2\rho }}\;. \end{array}$$

Now, we set *r*_1/4_=*r*_0_+*a*′, *r*_1/2_=*r*_0_+*b*′, *r*_3/4_=*r*_0_+*c*′ and *r*_1_=*r*_0_+*d*′. Note that, because the variables are correlated, the first ‘step’ (from *r*_0_ to *r*_1/4_) tends to be larger in magnitude than subsequent ‘steps’ (i.e. from *r*_1/4_ to *r*_1/2_, *r*_1/2_ to *r*_3/4_ or *r*_3/4_ to *r*_1_), which is why we have named this procedure ‘plateau’. This procedure might generate plausible *r*_*z*_ functions for a population in which worker sterility brings diminishing returns to colony productivity, where these diminishing returns happen to set in near $z=\frac{1}{4}$.

Note that both the ‘random noise’ and ‘plateau’ procedures tend to produce *r*_*z*_ functions that disadvantage single mating relative to double mating. For the ‘random noise’ procedure, this is because although the procedure is just as likely to produce a peak at $z=\frac{1}{2}$ (which would favour single mating) as at $z=\frac{1}{4}$ (which would favour double mating), workers at $z=\frac{1}{2}$ are typically ‘trading away’ more male production than workers at $z=\frac{1}{4}$ (because *p*_1/2_≥*p*_1/4_), yet, on average, they are receiving the same expected increase in productivity; hence, single mating is relatively disfavoured without a clear biological rationale. And as the ‘plateau’ procedure tends to produce colony efficiency functions with diminishing returns on worker sterility for colonies with $z>\frac{1}{4}$, it is much more likely to produce an *r*_*z*_ function with a relative peak at $z=\frac{1}{4}$ rather than a relative peak at $z=\frac{1}{2}$, thus relatively disfavouring the invasion of worker sterility under single mating without a clear biological rationale.

The third procedure, ‘random steps’, sets each point in *r*_*z*_ to the value of the previous point plus a random perturbation: *r*_1/4_=*r*_0_+*a*, *r*_1/2_=*r*_1/4_+*b*, *r*_3/4_=*r*_1/2_+*c* and *r*_1_=*r*_3/4_+*d*. This procedure might generate plausible *r*_*z*_ functions if each increase in worker sterility had a random increasing or decreasing effect on colony productivity. The fourth procedure, ‘increasing steps’, is similar, except steps are constrained to be positive: *r*_1/4_=*r*_0_+|*a*|, *r*_1/2_=*r*_1/4_+|*b*|, *r*_3/4_=*r*_1/2_+|*c*| and *r*_1_=*r*_3/4_+|*d*|. This procedure might generate plausible *r*_*z*_ functions if each increase in worker sterility added a random increase to colony productivity. The fifth procedure, ‘linear’, uses a single normal variate to establish a constant step size for *r*_*z*_: *r*_1/4_=*r*_0_+*a*, *r*_1/2_=*r*_1/4_+*a*, *r*_3/4_=*r*_1/2_+*a* and *r*_1_=*r*_3/4_+*a*. This procedure might generate plausible *r*_*z*_ functions if each increase in worker sterility had a consistent increasing or decreasing effect on colony productivity. For each of these new procedures, later points in *r*_*z*_ depend on earlier points, but there is no tendency for ‘steps’ between points in *r*_*z*_ to change in average magnitude, which arguably makes them less biased in favour of particular mating-number regimes than the old procedures.

In figure 3, we test each of these five procedures to see whether single or double mating is more favourable to the invasion (figure 3*b*) or equilibrium level of sterility (figure 3*c*), for recessive versus dominant sterility. The form of *p*_*z*_ we use (*p*_*z*_=*k*+(1−*k*)*z*, with *k*=0.2), chosen for comparison with the numerical experiments of Olejarz *et al.* [19], their table 1, prevents worker sterility from resulting in a net loss of nephews (see Spiteful worker sterility and policing, above). Beneath the bar charts in figure 3*b*, we show the percentage of experiments for which the exclusive invasion of sterility under either single or double mating occurred with an initially decelerating *r*_*z*_ (i.e. where *r*_1/2_−*r*_1/4_<*r*_1/4_−*r*_0_). Note that, for these values of *p*_*z*_, double mating only promotes the invasion of sterility relative to single mating when *r*_*z*_ is initially decelerating. In figure 3*c*, error bars show bootstrapped 95% confidence intervals for average worker sterility.

For the analysis presented in table 1, *r*_*z*_ functions are constructed using *σ*=0.25, and intermediate values (i.e. any *r*_*z*_ for $z\notin \{0,\frac{1}{4},\frac{1}{2},\frac{3}{4},1\}$) are linearly piecewise-interpolated between these points. For the same analysis, *p*_*z*_ functions are constructed using random variates as follows: *p*_0_ is drawn from a uniform distribution between 0 and 1; *p*_1/2_ is drawn from a uniform distribution between *p*_0_ and (*p*_0_+1)/2 (for non-spiteful worker sterility) or between (*p*_0_+1)/2 and 1 (for worker spite); *p*_1_=1; and all other values are linearly piecewise-interpolated between these three points.

### 4.4. Evolutionarily stable strategy analysis

By setting the left-hand side of condition (2.2) to zero, it is possible to find a convergence-stable point [12] for worker sterility. At these points, natural selection will not favour the invasion of an allele encoding either a small increase or a small decrease to worker sterility (i.e. convergence-stable points are stable to small perturbations); moreover, for a population playing a strategy that is close to a convergence-stable point, natural selection will favour the invasion of strategies between the population strategy and the convergence-stable point (i.e. convergence-stable states are reachable from nearby states). However, a convergence-stable point is only an ESS if *no* alternative allele can invade at this point. Therefore, in order to find a true ESS, we treat convergence-stable points as ‘candidate ESSs’, then use conditions (4.1) and (4.2) to determine whether any alternative allele can invade a population monomorphic for the candidate ESS under the appropriate regime of dominance or recessivity. If no alternative allele can invade, the candidate ESS is a true ESS. In figure 4, true ESSs are shown.

Note that it is possible for an ESS to *not*be convergence-stable, and this method will not identify such states. However, we are only interested in ESSs that are reachable, i.e. both convergence-stable and evolutionarily stable. Such strategies are called ‘continuously stable strategies’ (CSSs; [44]).

### 4.5. Demographically explicit ecological scenarios

In appendix B, we develop a general kin-selection model for the evolution of worker sterility. This analysis can be used to investigate a variety of ecological scenarios. Here, we present four such scenarios for the evolution of worker sterility.

#### 4.5.1. Scenario A. Workers’ sons replace queen’s sons

In this scenario, we assume that non-sterile workers replace the queen’s sons with their own sons, as in the model of Olejarz *et al.* [19]. Following these assumptions, we find that natural selection will favour an increase to worker sterility, *z*, when

4.3 $$\underset{\mathrm{sacrifice}\;\mathrm{effect}}{\underbrace{-\frac{1-p_{z}}{1-z}R_{\mathrm{son}}}}+\underset{\mathrm{efficiency}\;\mathrm{effect}}{\underbrace{\frac{r_{z}^{\prime }}{r_{z}}\left(R_{\mathrm{sis}}+p_{z}R_{\mathrm{bro}}+(1-p_{z})R_{\mathrm{neph}}\right)}}+\underset{\mathrm{male}\;\mathrm{production}\;\mathrm{effect}}{\underbrace{p_{z}^{\prime }R_{\mathrm{bro}}+\left(\frac{1-p_{z}}{1-z}-p_{z}^{\prime }\right)R_{\mathrm{neph}}}}>0,$$

where $R_{\mathrm{son}}=\frac{1}{2}$, *R*_neph_=(2+*n*)/8*n*, *R*_sis_=(1+*p*_*z*_)((2+*n*)/8*n*) and $R_{\mathrm{bro}}=\frac{1}{4}$. As explained in the main text, the left-hand side of condition (4.3) can be interpreted as the inclusive-fitness effect experienced by a worker who stops laying male eggs. The ‘sacrifice effect’ captures the direct cost of her sterility, in that she forfeits her relative share (1−*p*_*z*_)/(1−*z*) of all worker-laid males. The ‘efficiency effect’ captures her impact on colony efficiency, which increases by a relative amount *r*′_*z*_/*r*_*z*_, augmenting the production of her sisters and of colony-produced males, a proportion *p*_*z*_ of whom are her brothers and a proportion 1−*p*_*z*_ of whom are her nephews. And the ‘male production effect’ captures her impact on the proportion of male eggs produced by the queen versus workers: her relative gain of brothers is *p*′_*z*_, while her relative gain or loss of nephews exactly balances her forfeited sons and her gained brothers.

Similarly, natural selection favours an increase to the queen’s sex allocation, *x* (her proportion of resources allocated to daughters), when

4.4 $$\frac{1}{x}-\frac{1}{1-x}>0.$$

That is, natural selection favours an increased investment into daughters when $x<\frac{1}{2}$, and a decreased investment into daughters when $x>\frac{1}{2}$, such that an even sex ratio is favoured overall, regardless of worker sterility [45].

#### 4.5.2. Scenario B. Workers’ sons compete with all queen’s offspring

It is also possible to assume that, rather than only displacing the queen’s sons, workers’ sons compete with the queen’s sons and daughters equally. This scenario may apply if workers do not discern between fertilized and unfertilized eggs when they replace the queen’s eggs with their own; alternatively, it may apply if rather than replacing the queen’s eggs, the workers simply lay their eggs in the communal nest, and all queen-produced and worker-produced offspring have the same expected survival. Following these assumptions, we find that natural selection will favour an increase to worker sterility, *z*, when

4.5 $$\begin{array}{rl} & \underset{\mathrm{sacrifice}\;\mathrm{effect}}{\underbrace{-\frac{1-p_{z}}{1-z}R_{\mathrm{son}}}}+\underset{\mathrm{efficiency}\;\mathrm{effect}}{\underbrace{\frac{r_{z}^{\prime }}{r_{z}}(xp_{z}R_{\mathrm{sis}}+(1-x)p_{z}R_{\mathrm{bro}}+(1-p_{z})R_{\mathrm{neph}})}}\\ & \;\;+\underset{\mathrm{offspring}\;\mathrm{production}\;\mathrm{effect}}{\underbrace{xp_{z}^{\prime }R_{\mathrm{sis}}+(1-x)p_{z}^{\prime }R_{\mathrm{bro}}+\left(\frac{1-p_{z}}{1-z}-p_{z}^{\prime }\right)R_{\mathrm{neph}}}}>0, \end{array}$$

where *p*_*z*_ is the proportion of all offspring on the patch that are produced by the queen, $R_{\mathrm{son}}=\frac{1}{2}$, *R*_neph_=(2+*n*)/8*n*, *R*_sis_=((1+(1−2*x*)*p*_*z*_)/*xp*_*z*_)((2+*n*)/8*n*) and $R_{\mathrm{bro}}=\frac{1}{4}$. In this model, queen sex allocation alters the relative reproductive value of a female compared to that of a male, (1+(1−2*x*)*p*_*z*_)/*xp*_*z*_ (the product of the relative reproductive value of all females compared to that of all males, (1+(1−2*x*)*p*_*z*_)/(1−*xp*_*z*_), and the number of females relative to the number of males, (1−*xp*_*z*_)/*xp*_*z*_), which comes into the expression for *R*_sis_. Similarly to condition (4.3), the left-hand side of condition (4.5) can be interpreted as the inclusive-fitness effect experienced by a worker who stops laying male eggs. Here, the ‘sacrifice effect’ captures the direct cost of her sterility, in that she forfeits her relative share (1−*p*_*z*_)/(1−*z*) of all worker-laid males. The ‘efficiency effect’ captures her impact on colony efficiency, which increases by a relative amount *r*′_*z*_/*r*_*z*_, a proportion *xp*_*z*_ of which goes towards sisters, (1−*x*)*p*_*z*_ towards brothers, and 1−*p*_*z*_ towards nephews. And the ‘offspring production effect’ captures her impact on the proportion of eggs produced by the queen versus workers: her relative gain of sisters is *xp*′_*z*_, and her relative gain of brothers is (1−*x*)*p*′_*z*_, and hence her relative gain of nephews exactly balances her lost sons, less her gained brothers and sisters.

In this scenario, queen sex allocation is not independent of worker sterility. We find that natural selection favours an increase to the queen’s investment in daughters, *x*, when

4.6 $$\frac{1+p_{z}}{2x}-\frac{p_{z}}{1-x}>0;$$

hence, when all colony offspring are queen-laid (*p*_*z*_=1), the queen favours an even sex ratio ($x=\frac{1}{2}$), but as the proportion of colony offspring laid by workers increases, the queen favours an increasingly female-biased sex ratio. Specifically, the queen’s equilibrium sex ratio is x*=(1+*p*_*z*_)/(1+3*p*_*z*_), resulting in a population sex ratio of X*=*p*_*z*_(1+*p*_*z*_)/(1+3*p*_*z*_), which is male-biased for all *p*_*z*_<1.

#### 4.5.3. Scenario C. Worker sterility among claustral inbreeders

Here, we assume that the queen produces a first brood of female and male soldiers, who mate among themselves; the second brood of female and male dispersers is partly produced by the queen and partly produced by the soldiers, as in the gall-forming social thrips. For simplicity, we assume here that queens and soldiers produce an even sex ratio for the second brood, but allowing sex ratio evolution does not change the results qualitatively (not shown). Following these assumptions, we find that natural selection favours an increase to the sterility of female soldiers, *z*, when

4.7 $$\begin{array}{rl} & \underset{\mathrm{sacrifice}\;\mathrm{effect}}{\underbrace{-\frac{1-p_{z}}{1-z}(R_{\mathrm{dau}}+R_{\mathrm{son}})}}+\underset{\mathrm{efficiency}\;\mathrm{effect}}{\underbrace{\frac{r_{z}^{\prime }}{r_{z}}(p_{z}(R_{\mathrm{sis}}+R_{\mathrm{bro}})+(1-p_{z})(R_{\mathrm{niece}}+R_{\mathrm{neph}}))}}\\ & \;\;+\underset{\mathrm{offspring}\;\mathrm{production}\;\mathrm{effect}}{\underbrace{p_{z}^{\prime }(R_{\mathrm{sis}}+R_{\mathrm{bro}})+\left(\frac{1-p_{z}}{1-z}-p_{z}^{\prime }\right)(R_{\mathrm{niece}}+R_{\mathrm{neph}})}}>0, \end{array}$$

where, under haplodiploidy, *R*_dau_=(5+*p*_*z*_)/6, *R*_son_=(3+*p*_*z*_)/6, *R*_niece_=(3+6*n*+*p*_*z*_)/12*n*, *R*_neph_=(3+2*n*+*p*_*z*_)/12*n*, *R*_sis_=(3+2*n*+*p*_*z*_)/6*n* and $R_{\mathrm{bro}}=\frac{1}{3}$. Because this scenario does not require arrhenotokous parthenogenesis of males, it also applies to diploid populations. Under diploidy, *R*_dau_=*R*_son_=(11+*p*_*z*_)/16 and *R*_niece_=*R*_neph_=*R*_sis_=*R*_bro_=(1+*n*)/4*n* (figure 7*a*). Similarly to condition (4.5), the left-hand side of condition (4.7) can be interpreted as the inclusive-fitness effect experienced by a worker who stops laying male eggs; but in condition (4.7), the female worker’s ‘sacrifice effect’ involves giving up both daughters and sons; the ‘efficiency effect’ involves an increase in both niece and nephew production as well as sister and brother production; and the ‘offspring production effect’ involves the focal worker gaining both sisters and brothers, while her gain or loss of nieces and nephews balances her forfeited offspring and her gained siblings.

#### 4.5.4. Scenario D. The evolution of eusociality

Here, we assume that the queen produces and provisions a first brood of females, and then produces a second batch of female and male eggs. Each first-brood female can either disperse—leave the nest, mate, and produce female and male offspring on her own—or work—stay in the nest and help to raise the queen’s second-brood offspring without producing any offspring of her own. We assume that each worker can raise *b* siblings, on average, in her natal nest, and that each disperser can raise *b*(1−*c*) offspring, on average, in her newly founded nest, where *c* represents the cost of dispersal; and, additionally, that workers may synergistically or antagonistically interact according to the parameter *s*, such that if the total number of female workers is *Kz*, then in total workers can raise *Kzb*(1+*sz*) of the queen’s second-brood offspring. This model is conceptually similar to the one considered by Boomsma [8–10] for the evolution of eusociality. Following these assumptions, we find that natural selection will favour an increase to worker sterility, *z*, when

4.8 $$\underset{\mathrm{sacrifice}\;\mathrm{effect}}{\underbrace{-b(1-c)(R_{\mathrm{dau}}+R_{\mathrm{son}})}}+\underset{\mathrm{efficiency}\;\mathrm{effect}}{\underbrace{b(1+2sz)(R_{\mathrm{sis}}+R_{\mathrm{bro}})}}>0,$$

where $R_{\mathrm{dau}}=R_{\mathrm{son}}=\frac{1}{2}$, *R*_sis_=(2+*n*)/4*n* and $R_{\mathrm{bro}}=\frac{1}{4}$. As with scenario C, this scenario also applies to diploid populations; under diploidy, $R_{\mathrm{dau}}=R_{\mathrm{son}}=\frac{1}{2}$ and *R*_sis_=*R*_bro_=(1+*n*)/4*n* (figure 7*b*). When *z*=0, this condition reduces to

$$c>\frac{n-1}{2n}$$

under both haplodiploidy and diploidy; that is, under strict monogamy (*n*=1), any marginal benefit of rearing siblings rather than offspring (for example, any non-zero cost of dispersal, mating or nest founding) suffices to favour the invasion of sterile workers, regardless of the level of worker synergy, *s*; but with any level of multiple mating (*n*>1), a threshold dispersal cost of at least (*n*−1)/2*n* is required for natural selection to favour the invasion of sterile workers (figures 6*d* and 7*b*). In other words, only marginal efficiency gains are needed for worker sterility to invade under strict monogamy [8–10].

#### 4.5.5 Explicit forms for *r*_*z*_ and *p*_*z*_

Scenarios A, B and C above are independent of the particular *r*_*z*_ and *p*_*z*_ functions used. However, for preparing figures 6–8, we used the explicit forms

$$\begin{array}{rl} r_{z} & \;=1+bz+sz^{2}\;\text{and}\\ p_{z} & \;=\frac{1}{1+k(1-z)}. \end{array}$$

The *r*_*z*_ function above has three components: a baseline efficiency of 1; *bz*, representing a linear fitness benefit for each sterile worker; and *sz*^2^, representing an ‘interaction effect’ of worker sterility. We use the parameter *s* to examine scenarios where multiple sterile workers result in either accelerating (*s*>0) or diminishing returns (*s*<0) to colony productivity.

The *p*_*z*_ function given above corresponds to a model in which the queen and *k*(1−*z*) reproductive workers each take an equal share of offspring production. Alternatively, *k* can capture not only the total number of workers but also their ability to control offspring production relative to the queen; for example, halving *k* could represent either a halving in the number of workers or a halving of their relative ability to control offspring production, keeping the number of workers constant.

A function of this form can also model more complicated demographic processes: for example, if we assume that there are *N* workers, each of whom replaces a random egg with their own at rate *W*, while the queen can replace a worker’s egg with her own at rate *Q*, then the form above gives the proportion of eggs produced by the queen at equilibrium when *k*=*NW*/*Q*. In models where worker-laid and queen-laid individuals compete equally, regardless of their sex, production of eggs and replacement of eggs will often be equivalent processes: that is, the form given above for *p*_*z*_ also holds if workers, rather than replacing the queen’s eggs, simply lay their own eggs in the communal nest without replacement. In that case, the *r*_*z*_ function would capture both the overall production and survival of eggs.

#### 4.5.6. Stable level of sterility

For figures 6 and 7, we determine the convergence-stable point [12] for sterility by numerically integrating the selection gradients for sterility and sex allocation (left-hand sides of conditions (4.3)–(4.8)). First, we set the sex ratio to $x=\bar{x}=\frac{1}{2}$ and allow it to evolve in the absence of worker sterility ($Z=z=\bar{z}=0$) until it reaches its equilibrium value. Then, we allow both the sex ratio and sterility to co-evolve, until equilibrium is reached for both traits.

### 4.6. Stochastic individual-based model

To verify the results of our kin-selection analysis (figures 4, 6 and 7), we implemented a stochastic individual-based model (figures 5 and 8) in C++. Here, each individual comprises a locus encoding their breeding value for worker sterility, *Z*. The locus comprises one or two genes, depending on whether the individual is haploid or diploid, and each gene is represented by a real number *γ*∈[0,1]. Breeding values are determined by averaging genic values: hence, a haploid individual with genotype *γ* has breeding value *Z*=*γ*, while a diploid individual with genotype *γ*_1_,*γ*_2_ has breeding value *Z*=(*γ*_1_+*γ*_2_)/2.

At the beginning of each generation, *M* mated females each produce *K* female workers on their home patch. Each worker has a probability *Z* of being sterile. The patch average sterility *z* determines the colony productivity *r*_*z*_ and the proportion of males produced by the queen *p*_*z*_. The next generation of breeders is then produced: first, a patch is randomly selected from the population with probability proportional to its colony efficiency, *r*_*z*_, and a female is produced by the queen on that patch; then, another *n* patches are randomly selected with replacement, with probability proportional to their colony efficiency, and each of these *n* patches produces a male (from the queen with probability *p*_*z*_, or from a random reproductive worker on that patch with probability 1−*p*_*z*_); the female mates with these *n* males, and this process is performed *M* times, at which point all the *M* mated females replace the foundresses of existing patches. All other individuals on each patch die, returning the population to the beginning of the life cycle.

Simulations start with a monomorphic population in which all *γ*=0, and hence *Z*=0 for each individual. A gene in a newly produced individual has a 1% probability of mutating, in which case its genic value changes from *γ* to $\gamma^{\prime }=\max(0,\min(\gamma +\delta ,1))$, where *δ* is drawn from a normal distribution with mean 0 and standard deviation 0.01. We validated this stochastic individual-based model by using it to verify the analytical conditions of Olejarz *et al.* [19], not shown.

For figure 8, we make the following assumptions. The mating number *n* is either fixed at 1 (species 1–10) or drawn randomly from 1 to 5 (species 11–60). Each species’ *p*_*z*_ function uses the form *p*_*z*_=1/(1+*k*(1−*z*)) (see Explicit forms for *r*_*z*_ and *p*_*z*_, above), where *k* for each species is drawn randomly from 1 to 5. Finally, each species’ *r*_*z*_ function is of the form *r*_*z*_=1+*bz*+*sz*^2^ (see Explicit forms for *r*_*z*_ and *p*_*z*_, above), with *b* and *s* chosen such that *r*_1_ follows a uniform distribution between 1.5 and 2.5 and such that the slope *r*′_0_ is between 50% and 150% of the slope of the line between (*z*=0,*r*_0_) and (*z*=1,*r*_1_). In this way, the colony productivity function is equally likely to be concave or convex.

## Data accessibility

Data and code are deposited at Dryad: https://doi.org/10.5061/dryad.gt8b5 [46].

## Appendix A: Explicit population-genetics analysis

Here, we analyse the invasion of a sterility allele into a wild-type population. The population is initially monomorphic for an allele *A* encoding sterility with penetrance 0≤*u*≤1, and a rare mutant allele *a* is introduced which encodes sterility with penetrance 0≤*v*≤1. Throughout, we closely follow the approach of Olejarz *et al.* [19], whose analysis is equivalent to ours with the assumptions that *u* and *v* are restricted to either 0 or 1.

We denote colony types by the genotype of the queen and the genotypes of her mating partners. Hence, *X*_*AA*,*m*_ is the frequency of colonies with an *AA* queen, *m* mutant (*a*) males and *n*−*m* wild-type (*A*) males; similarly for *X*_*Aa*,*m*_ and *X*_*aa*,*m*_. At any given time step, we also keep track of the number of reproductive females of each genotype—*x*_*AA*_, *x*_*Aa*_ and *x*_*aa*_—and the number of reproductive males of each genotype—*y*_*A*_ and *y*_*a*_. Matings between reproductives lead to the establishment of new colonies; hence, the evolutionary dynamics of colony types are captured by

A 1 X˙AA,m =xAA(nm)yAn−myam−ϕXAA,m,X˙Aa,m =xAa(nm)yAn−myam−ϕXAa,mandX˙aa,m =xaa(nm)yAn−myam−ϕXaa,m.

That is, the rate of establishment of new *AA*,*m* colonies is proportional to the frequency of reproductive *AA* females, multiplied by their probability of mating with exactly *n*−*m* wild-type males and *m* mutant males; similarly for *Aa*,*m* and *aa*,*m* colonies.

The death rate of existing colonies, *ϕ*, is defined as

A 2 $$\phi =(x_{AA}+x_{Aa}+x_{aa}){\left(y_{A}+y_{a}\right)}^{n},$$

in order to enforce a density constraint, namely

A 3 $$\sum_{m=0}^{n}(X_{AA,m}+X_{Aa,m}+X_{aa,m})=1.$$

**A.1. Reproductive offspring if the mutant allele is dominant**

When the mutant allele is dominant, the production of each type of reproductive female (*x*_*AA*_, *x*_*Aa*_, *x*_*aa*_) and male (*y*_*A*_, *y*_*a*_) is

A 4 xAA =∑m=0n{n−mnr((n−m)u+mv)/nXAA,m+n−m2nr((n−m)u+(n+m)v)/2nXAa,m},xAa =∑m=0n{mnr((n−m)u+mv)/nXAA,m+12r((n−m)u+(n+m)v)/2nXAa,m+n−mnrvXaa,m},xaa =∑m=0n{m2nr((n−m)u+(n+m)v)/2nXAa,m+mnrvXaa,m},yA =∑m=0n{(p((n−m)u+mv)/n+((n−m)/n)(1−u)+(1/2)(m/n)(1−v)((n−m)/n)(1−u)+(m/n)(1−v)(1−p((n−m)u+mv)/n))× r((n−m)u+mv)/nXAA,m+(12p((n−m)u+(n+m)v)/2n+((n−m)/2n)(1−u)+(1/4)(1−v)((n−m)/2n)(1−u)+(1/2)(1−v)+(m/2n)(1−v)×12(1−p((n−m)u+(n+m)v)/2n))r((n−m)u+(n+m)v)/2nXAa,m+(12n−mn)(1−pv)rvXaa,m}andya =∑m=0n{((1/2)(m/n)(1−v)((n−m)/n)(1−u)+(m/n)(1−v)(1−p((n−m)u+mv)/n))r((n−m)u+mv)/nXAA,m+(12p((n−m)u+(n+m)v)/2n+(1/4)(1−v)+(m/2n)(1−v)((n−m)/2n)(1−u)+(1/2)(1−v)+(m/2n)(1−v)×12(1−p((n−m)u+(n+m)v)/2n))r((n−m)u+(n+m)v)/2nXAa,m+(pv+(12n−mn+mn)(1−pv))rvXaa,m}.

These equations can be understood as follows. First, note that in an *AA*,*m* colony, a fraction *z*=((*n*−*m*)/*n*)*u*+(*m*/*n*)*v*=((*n*−*m*)*u*+*mv*)/*n* of workers will be sterile (*AA* workers with probability *u*, and *Aa* workers with probability *v*); in an *Aa*,*m* colony, a fraction $z=((n-m)/2n)u+\frac{1}{2}v+(m/2n)v=((n-m)u+(n+m)v)/2n$ of workers will be sterile (*AA* workers with probability *u*, and *Aa* and *aa* workers with probability *v*); and in an *aa*,*m* colony, a fraction *z*=((*n*−*m*)/*n*)*v*+(*m*/*n*)*v*=*v* of workers will be sterile (*Aa* and *aa* workers with probability *v*). That is why these values of *z* as subscripts to the *r*_*z*_ and *p*_*z*_ functions are always associated, above, with their associated colony frequencies, *X*_*AA*,*m*_, *X*_*Aa*,*m*_ and *X*_*aa*,*m*_, respectively.

For female reproductives, each separate term within the curly braces above combines three elements; we will take the first term in curly braces in the *x*_*AA*_ line,

$$\frac{n-m}{n}r_{(nu+m(v-u))/n}X_{AA,m},$$

as an example. The three elements are the frequency of a given colony type (i.e. *X*_*AA*,*m*_); the productivity of that colony type, as a function of the fraction of sterile workers within colonies of that type (i.e. *r*_(*nu*+*m*(*v*−*u*))/*n*_); and the fraction of females and/or males produced by that colony type with the corresponding genotype (i.e. a fraction (*n*−*m*)/*n* of females produced in *AA*,*m* colonies have genotype *AA*, which is why they add to the quantity *x*_*AA*_). Each term within equation (A 12) can be broken down in this way.

Accordingly, the production of female reproductives can be understood as follows: *AA*,*m* colonies produce (*n*−*m*)/*n* *AA* females and *m*/*n* *Aa* females; *Aa*,*m* colonies produce (*n*−*m*)/2*n* *AA* females, $\frac{1}{2}$ *Aa* females and *m*/2*n* *aa* females; and *aa*,*m* colonies produce (*n*−*m*)/*n* *Aa* females and *m*/*n* *aa* females.

Male production is more complicated, because both queens and workers produce males, but the principle is the same. We will take the first term in curly braces in the *y*_*A*_ line,

$$\begin{array}{rl} & \left(p_{((n-m)u+mv)/n}+\frac{\left((n-m)/n)(1-u)+(1/2)(m/n)(1-v\right)}{\left((n-m)/n)(1-u)+(m/n)(1-v\right)}\left(1-p_{((n-m)u+mv)/n}\right)\right)\\ & \;\;\times r_{((n-m)u+mv)/n}X_{AA,m}, \end{array}$$

as an example. Here, the overall productivity of *AA*,*m* colonies (i.e. *r*_((*n*−*m*)*u*+*mv*)/*n*_*X*_*AA*,*m*_) goes towards the production of both the queen’s sons and workers’ sons. In particular, the queen is *AA*, so all her sons have genotype *A*, and the queen produces a fraction *p*_((*n*−*m*)*u*+*mv*)/*n*_ of males in the colony. Simultaneously, the workers—whose sons comprise a fraction 1−*p*_((*n*−*m*)*u*+*mv*)/*n*_ of colony male production—are (*n*−*m*)/*n* *AA* and *m*/*n* *Aa*; in the former group, workers are reproductive with probability 1−*u*, while in the latter group, workers are reproductive with probability 1−*v*; and all the sons of the first group will be *A*, while only half of the sons of the second group will be *A*. Hence, overall, a fraction (((*n*−*m*)/*n*)(1−*u*)+(1/2)(*m*/*n*)(1−*v*)((*n*−*m*)/*n*)(1−*u*)+(*m*/*n*)(1−*v*))(1−*p*_((*n*−*m*)*u*+*mv*)/*n*_) of males produced in *AA*,*m* colonies are *A* males produced by workers. Note that the expressions for *y*_*A*_ and *y*_*a*_ can be further simplified, but we have left them in the form above to maximize clarity.

Accordingly, the production of male reproductives can be understood as follows. In *AA*,*m* colonies, the queen’s sons are all *A*; all of the sons of *AA* workers and half of the sons of *Aa* workers are *A*, while the other half of the sons of *Aa* workers are *a*. In *Aa*,*m* colonies, the queen’s sons are half *A* and half *a*; all of the sons of *AA* workers and half of the sons of *Aa* workers are *A*, while the other half of the sons of *Aa* workers and all of the sons of *aa* workers are *a*. Finally, in *aa*,*m* colonies, the queen’s sons are all *a*; half of the sons of *Aa* workers are *A*, while the other half of the sons of *Aa* workers and all the sons of *aa* workers are *a*.

**A.2. Reproductive offspring if the mutant allele is recessive**

Along similar principles, when the mutant allele is recessive, the production of each type of reproductive female and male is

A 5 xAA =∑m=0n{n−mnruXAA,m+n−m2nr((2n−m)u+mv)/2nXAa,m},xAa =∑m=0n{mnruXAA,m+12r((2n−m)u+mv)/2nXAa,m+n−mnr((n−m)u+mv)/nXaa,m},xaa =∑m=0n{m2nr((2n−m)u+mv)/2nXAa,m+mnr((n−m)u+mv)/nXaa,m},yA =∑m=0n{(pu+(n−mn+12mn)(1−pu))ruXAA,m+(12p((2n−m)u+mv)/2n+((n−m)/2n)(1−u)+(1/4)(1−u)((n−m)/2n)(1−u)+(1/2)(1−u)+(m/2n)(1−v)×12(1−p((2n−m)u+mv)/2n))r((2n−m)u+mv)/2nXAa,m+(12 n−mn(1−u)n−mn(1−u)+mn(1−v))(1−p((n−m)u+mv)/n)r((n−m)u+mv)/nXaa,m}andya =∑m=0n{(12mn)(1−pu)ruXAA,m+(12p((2n−m)u+mv)/2n+(1/4)(1−u)+(m/2n)(1−v)((n−m)/2n)(1−u)+(1/2)(1−u)+(m/2n)(1−v)×12(1−p((2n−m)u+mv)/2n))r((2n−m)u+mv)/2nXAa,m+(p((n−m)u+mv)/n+((1/2)((n−m)/n)(1−u)+(m/n)(1−v)((n−m)/n)(1−u)+(m/n)(1−v))×12(1−p((n−m)u+mv)/n))r((n−m)u+mv)/nXaa,m}.

These equations can be understood similarly to equation (A 12); in fact, they are identical, except for two general changes. First, the subscripts to *r*_*z*_ and *p*_*z*_ are different, because the mutant allele is recessive instead of dominant, which results in different proportions of sterile workers in colonies of each type: in an *AA*,*m* colony, a fraction *z*=((*n*−*m*)/*n*)*u*+(*m*/*n*)*u*=*u* of workers will be sterile; in an *Aa*,*m* colony, a fraction $z=((n-m)/2n)u+\frac{1}{2}u+(m/2n)v=((2n-m)u+mv)/2n$ of workers will be sterile; and in an *aa*,*m* colony, a fraction *z*=((*n*−*m*)/*n*)*u*+(*m*/*n*)*v*=((*n*−*m*)*u*+*mv*)/*n* of workers will be sterile. Second, because of these differing proportions of sterile workers, the production of sons by workers is different, so the coefficients of 1−*p*_*z*_ in the fourth and fifth lines are different.

**A.3. Condition for invasion of a dominant mutant sterility allele**

Continuing to follow the approach of Olejarz *et al.* [19]: for a dominant mutant sterility allele, whether the allele increases in frequency from rarity is governed by the behaviour of *AA*,0, *AA*,1 and *Aa*,0 colonies. Colony types with more copies of the mutant allele are rarer, and hence will have a negligible effect on invasion. Therefore, from equation (A 9), we need only consider

A 6 $$\begin{array}{rl} {\dot{X}}_{AA,0} & \;=x_{AA}y_{A}^{n}-\phi X_{AA,0},\\ {\dot{X}}_{AA,1} & \;=nx_{AA}y_{A}^{n-1}y_{a}-\phi X_{AA,1}\\ \text{and}\;{\dot{X}}_{Aa,0} & \;=x_{Aa}y_{A}^{n}-\phi X_{Aa,0}. \end{array}$$

We start with a wild-type population (*X*_*AA*,0_=1) and introduce a small perturbation of magnitude *ϵ*≪1. Considering the density constraint (equation (A 11)), and only keeping terms up to order *ϵ*, this gives

A 7 $$\begin{array}{rl} X_{AA,0} & \;=1-\epsilon (\delta_{AA,1}^{\left(1\right)}+\delta_{Aa,0}^{\left(1\right)})-O(\epsilon^{2})\\ X_{AA,1} & \;=\epsilon \delta_{AA,1}^{\left(1\right)}+O(\epsilon^{2})\\ \text{and}\;X_{Aa,0} & \;=\epsilon \delta_{Aa,0}^{\left(1\right)}+O(\epsilon^{2}), \end{array}$$

which implies that

A 8 $$\begin{array}{rl} {\dot{X}}_{AA,1} & \;=\epsilon {\dot{\delta }}_{AA,1}^{\left(1\right)}+O(\epsilon^{2})\\ \text{and}\;{\dot{X}}_{Aa,0} & \;=\epsilon {\dot{\delta }}_{Aa,0}^{\left(1\right)}+O(\epsilon^{2}). \end{array}$$

Substituting (A 15) into (A 12), and keeping terms only up to order *ϵ*, gives

A 9 $$\begin{array}{rl} x_{AA} & \;=r_{u}+\epsilon \left(-r_{u}(\delta_{AA,1}^{\left(1\right)}+\delta_{Aa,0}^{\left(1\right)})+\frac{n-1}{n}r_{((n-1)u+v)/n}\delta_{AA,1}^{\left(1\right)}+\frac{1}{2}r_{(u+v)/2}\delta_{Aa,0}^{\left(1\right)}\right)+O(\epsilon^{2}),\\ x_{Aa} & \;=\epsilon \left(\frac{1}{n}r_{((n-1)u+v)/n}\delta_{AA,1}^{\left(1\right)}+\frac{1}{2}r_{(u+v)/2}\delta_{Aa,0}^{\left(1\right)}\right)+O(\epsilon^{2}),\\ x_{aa} & \;=0+O(\epsilon^{2}),\\ y_{A} & \;=r_{u}+\epsilon \left(\begin{array}{l} -r_{u}(\delta_{AA,1}^{\left(1\right)}+\delta_{Aa,0}^{\left(1\right)})+\frac{2n(1-u)+2u-1-v+(1-v)p_{((n-1)u+v)/n}}{2(n(1-u)-(v-u))}\\ \;r_{((n-1)u+v)/n}\delta_{AA,1}^{\left(1\right)}+\frac{3-2u-v-(1-u)p_{(u+v)/2}}{2(2-u-v)}r_{(u+v)/2}\delta_{Aa,0}^{\left(1\right)} \end{array}\right)+O(\epsilon^{2})\\ \text{and}\;y_{a} & \;=\epsilon \left(\frac{\left(1-v)(1-p_{((n-1)u+v)/n}\right)}{2(n(1-u)-(v-u))}r_{((n-1)u+v)/n}\delta_{AA,1}^{\left(1\right)}+\frac{1-v+(1-u)p_{(u+v)/2}}{2(2-u-v)}r_{(u+v)/2}\delta_{Aa,0}^{\left(1\right)}\right)+O(\epsilon^{2}). \end{array}$$

Finally, substituting (A 10), (A 16) and (A 17) into (A 14) and discarding powers of *ϵ*^2^ or higher gives

$$\begin{array}{rl} \epsilon {\dot{\delta }}_{AA,1} & \;=\epsilon r_{u}^{n}(-r_{u}\delta_{AA,1}^{\left(1\right)}+n(\frac{\left(1-v)(1-p_{((n-1)u+v)/n}\right)}{2(n(1-u)-(v-u))}r_{((n-1)u+v)/n}\delta_{AA,1}^{\left(1\right)}\\ & \;\;+\frac{1-v+(1-u)p_{(u+v)/2}}{2(2-u-v)}r_{(u+v)/2}\delta_{Aa,0}^{\left(1\right)}))\\ \epsilon {\dot{\delta }}_{Aa,0} & \;=\epsilon r_{u}^{n}\left(\frac{1}{n}r_{((n-1)u+v)/n}\delta_{AA,1}^{\left(1\right)}-r_{u}\delta_{Aa,0}^{\left(1\right)}+\frac{1}{2}r_{(u+v)/2}\delta_{Aa,0}^{\left(1\right)}\right). \end{array}$$

This can be rewritten in matrix form as

$$\begin{array}{rl} \left[\begin{array}{c} {\dot{\delta }}_{AA,1}^{\left(1\right)}\\ {\dot{\delta }}_{Aa,0}^{\left(1\right)} \end{array}\right] & \;=\left[\begin{array}{cc} r_{u}^{n}\left(-r_{u}+n\frac{\left(1-v)(1-p_{((n-1)u+v)/n}\right)}{2(n(1-u)-(v-u))}r_{((n-1)u+v)/n}\right) & r_{u}^{n}n\frac{1-v+(1-u)p_{(u+v)/2}}{2(2-u-v)}r_{(u+v)/2}\\ r_{u}^{n}\frac{1}{n}r_{((n-1)u+v)/n} & r_{u}^{n}\left(-r_{u}+\frac{1}{2}r_{(u+v)/2}\right) \end{array}\right]\\ & \;\;\times \left[\begin{array}{c} \delta_{AA,1}^{\left(1\right)}\\ \delta_{Aa,0}^{\left(1\right)} \end{array}\right]. \end{array}$$

If the dominant eigenvalue of the above matrix is greater than zero, then a dominant sterility allele with penetrance *v* can invade a population monomorphic for sterility with penetrance *u*. This condition, after simplification, is

A 10 $$\frac{r_{(u+v)/2}}{r_{u}}\left(\begin{array}{l} 1+\left(\frac{(1-u)p_{(u+v)/2}}{2-u-v}+\frac{\left(1-v)((2-n)(u-v)+n(2-u-v)p_{((n-1)u+v)/n}\right)}{2(n(1-u)+u-v)(2-u-v)}\right)\frac{r_{((n-1)u+v)/n}}{r_{u}}\\ \;+\frac{n(1-v)(1-p_{((n-1)u+v)/n})}{n(1-u)+u-v}\;\frac{r_{((n-1)u+v)/n}}{r_{(u+v)/2}} \end{array}\right)>2.$$

**A.4. Condition for invasion of a recessive mutant sterility allele**

For a recessive mutant sterility allele, whether the allele increases in frequency from rarity is governed by the behaviour of *AA*,0, *AA*,1, *Aa*,0, *AA*,2, *Aa*,1 and *aa*,0 colonies. Colony types with more copies of the mutant allele are rarer, and hence will have a negligible effect on invasion. Therefore, from equation (A 9), we need only consider

A 11 $$\begin{array}{rl} {\dot{X}}_{AA,0} & \;=x_{AA}y_{A}^{n}-\phi X_{AA,0,}\\ {\dot{X}}_{AA,1} & \;=nx_{AA}y_{A}^{n-1}y_{a}-\phi X_{AA,1,}\\ {\dot{X}}_{Aa,0} & \;=x_{Aa}y_{A}^{n}-\phi X_{Aa,0,}\\ {\dot{X}}_{AA,2} & \;=\frac{n(n-1)}{2}x_{AA}y_{A}^{n-2}y_{a}^{2}-\phi X_{AA,2},\\ {\dot{X}}_{Aa,1} & \;=nx_{Aa}y_{A}^{n-1}y_{a}-\phi X_{Aa,1}\\ \text{and}\;{\dot{X}}_{aa,0} & \;=x_{aa}y_{A}^{n}-\phi X_{aa,0}. \end{array}$$

We start with a wild-type population (*X*_*AA*,0_=1) and introduce a small perturbation of magnitude *ϵ*≪1. Considering the density constraint (equation (A 11)), and only keeping terms up to order *ϵ*^2^ (because terms of order *ϵ* alone are not sufficient to determine whether the recessive allele invades), this gives

A 12 $$\begin{array}{rl} X_{AA,0} & \;=1-\epsilon \delta_{AA,0}^{\left(1\right)}-\epsilon^{2}\delta_{AA,0}^{\left(2\right)}-O(\epsilon^{3}),\\ & \;=1-\epsilon (\delta_{AA,1}^{\left(1\right)}+\delta_{Aa,0}^{\left(1\right)})-\epsilon^{2}(\delta_{AA,1}^{\left(2\right)}+\delta_{Aa,0}^{\left(2\right)}+\delta_{AA,2}^{\left(2\right)}+\delta_{Aa,1}^{\left(2\right)}+\delta_{aa,0}^{\left(2\right)})-O(\epsilon^{3})\\ X_{AA,1} & \;=\epsilon \delta_{AA,1}^{\left(1\right)}+\epsilon^{2}\delta_{AA,1}^{\left(2\right)}+O(\epsilon^{3}),\\ X_{Aa,0} & \;=\epsilon \delta_{Aa,0}^{\left(1\right)}+\epsilon^{2}\delta_{Aa,0}^{\left(2\right)}+O(\epsilon^{3}),\\ X_{AA,2} & \;=\epsilon^{2}\delta_{AA,2}^{\left(2\right)}+O(\epsilon^{3}),\\ X_{Aa,1} & \;=\epsilon^{2}\delta_{Aa,1}^{\left(2\right)}+O(\epsilon^{3})\\ \text{and}\;X_{aa,0} & \;=\epsilon^{2}\delta_{aa,0}^{\left(2\right)}+O(\epsilon^{3}), \end{array}$$

which implies that

A 13 $$\begin{array}{rl} {\dot{X}}_{AA,0} & \;=-\epsilon {\dot{\delta }}_{AA,0}^{\left(1\right)}-\epsilon^{2}{\dot{\delta }}_{AA,0}^{\left(2\right)}-O(\epsilon^{3}),\\ & \;=-\epsilon ({\dot{\delta }}_{AA,1}^{\left(1\right)}+{\dot{\delta }}_{Aa,0}^{\left(1\right)})-\epsilon^{2}({\dot{\delta }}_{AA,1}^{\left(2\right)}+{\dot{\delta }}_{Aa,0}^{\left(2\right)}+{\dot{\delta }}_{AA,2}^{\left(2\right)}+{\dot{\delta }}_{Aa,1}^{\left(2\right)}+{\dot{\delta }}_{aa,0}^{\left(2\right)})-O(\epsilon^{3})\\ {\dot{X}}_{AA,1} & \;=\epsilon {\dot{\delta }}_{AA,1}^{\left(1\right)}+\epsilon^{2}{\dot{\delta }}_{AA,1}^{\left(2\right)}+O(\epsilon^{3}),\\ {\dot{X}}_{Aa,0} & \;=\epsilon {\dot{\delta }}_{Aa,0}^{\left(1\right)}+\epsilon^{2}{\dot{\delta }}_{Aa,0}^{\left(2\right)}+O(\epsilon^{3}),\\ {\dot{X}}_{AA,2} & \;=\epsilon^{2}{\dot{\delta }}_{AA,2}^{\left(2\right)}+O(\epsilon^{3}),\\ {\dot{X}}_{Aa,1} & \;=\epsilon^{2}{\dot{\delta }}_{Aa,1}^{\left(2\right)}+O(\epsilon^{3})\\ \text{and}\;{\dot{X}}_{aa,0} & \;=\epsilon^{2}{\dot{\delta }}_{aa,0}^{\left(2\right)}+O(\epsilon^{3}). \end{array}$$

Substituting equation (A 20) into equation (A 13), and keeping terms only up to order *ϵ*^2^, gives

A 14 $$\begin{array}{r} \begin{array}{rl} x_{AA} & \;=r_{u}+\epsilon \left(-\frac{1}{n}r_{u}\delta_{AA,1}^{\left(1\right)}-\frac{1}{2}r_{u}\delta_{Aa,0}^{\left(1\right)}\right)\\ & \;\;+\epsilon^{2}\left(\begin{array}{l} -\frac{1}{n}r_{u}\delta_{AA,1}^{\left(2\right)}-\frac{1}{2}r_{u}\delta_{Aa,0}^{\left(2\right)}-\frac{2}{n}r_{u}\delta_{AA,2}^{\left(2\right)}\\ \;+\left(\frac{n-1}{2n}r_{((2n-1)u+v)/2n}-r_{u}\right)\delta_{Aa,1}^{\left(2\right)}-r_{u}\delta_{aa,0}^{\left(2\right)} \end{array}\right)\;+O(\epsilon^{3}),\\ x_{Aa} & \;=\epsilon \left(\frac{1}{n}r_{u}\delta_{AA,1}^{\left(1\right)}+\frac{1}{2}r_{u}\delta_{Aa,0}^{\left(1\right)}\right)\\ & \;\;+\epsilon^{2}\left(\frac{r_{u}}{n}\delta_{AA,1}^{\left(2\right)}+\frac{1}{2}r_{u}\delta_{Aa,0}^{\left(2\right)}+\frac{2}{n}r_{u}\delta_{AA,2}^{\left(2\right)}+\frac{1}{2}r_{((2n-1)u+v)/2n}+r_{u}\delta_{aa,0}^{\left(2\right)}\right)+O(\epsilon^{3}),\\ x_{aa} & \;=\epsilon^{2}\left(\frac{1}{2n}r_{((2n-1)u+v)/2n}\delta_{Aa,1}^{\left(2\right)}\right)+O(\epsilon^{3}),\\ y_{A} & \;=r_{u}+\epsilon \left(-\frac{1-p_{u}}{2n}r_{u}\delta_{AA,1}^{\left(1\right)}-\frac{1+p_{u}}{4}r_{u}\delta_{Aa,0}^{\left(1\right)}\right)\\ & \;\;+\epsilon^{2}\left(\begin{array}{l} -\frac{2-p_{u}}{2n}r_{u}\delta_{AA,1}^{\left(2\right)}-\frac{1+p_{u}}{4}r_{u}\delta_{Aa,0}^{\left(2\right)}-\frac{1-p_{u}}{n}r_{u}\delta_{AA,2}^{\left(2\right)}\\ \;+\left(\frac{(3n-2)(1-u)+(2-n(1-u)-u-v)p_{((2n-1)u+v)/2n}}{2n(1-u)-(v-u)}r_{((2n-1)u+v)/2n}-r_{u}\right)\\ \;\times \delta_{Aa,1}^{\left(2\right)}-\frac{1+p_{u}}{2}r_{u}\delta_{aa,0}^{\left(2\right)} \end{array}\right)\\ & \;\;+O(\epsilon^{3})\\ \mathrm{and}\;y_{a} & \;=\epsilon \left(\frac{1-p_{u}}{2n}r_{u}\delta_{AA,1}^{\left(1\right)}+\frac{1+p_{u}}{4}r_{u}\delta_{Aa,0}^{\left(1\right)}\right)\\ & \;\;+\epsilon^{2}\left(\begin{array}{l} \frac{1-p_{u}}{2n}r_{u}\delta_{AA,1}^{\left(2\right)}+\frac{1+p_{u}}{4}r_{u}\delta_{Aa,0}^{\left(2\right)}+\frac{1-p_{u}}{n}r_{u}\delta_{AA,2}^{\left(2\right)}\\ \;+\frac{2+n(1-u)-2v-(2-n(1-u)-u-v)p_{((2n-1)u+v)/2n}}{2(2n(1-u)-(v-u))}\\ \;\times r_{((2n-1)u+v)/2n}\delta_{Aa,1}^{\left(2\right)}+\frac{1+p_{u}}{2}r_{u}\delta_{aa,0}^{\left(2\right)} \end{array}\right)+O(\epsilon^{3}). \end{array} \end{array}$$

Substituting equations (A 10), (A 21) and (A 22) into equation (A 19) and discarding powers of *ϵ*^2^ or higher gives, in matrix form,

$$\left[\begin{array}{c} {\dot{\delta }}_{AA,1}^{\left(1\right)}\\ {\dot{\delta }}_{Aa,0}^{\left(1\right)} \end{array}\right]=r_{u}^{n+1}\left[\begin{array}{cc} -\frac{1+p_{u}}{2} & \frac{n(1+p_{u})}{4}\\ \frac{1}{n} & -\frac{1}{2} \end{array}\right]\left[\begin{array}{c} \delta_{AA,1}^{\left(1\right)}\\ \delta_{Aa,0}^{\left(1\right)} \end{array}\right].$$

The dominant eigenvalue is 0, and its corresponding eigenvector is $\left[\begin{array}{c} n\\ 2 \end{array}\right]$, which gives

A 15 $$\begin{array}{rl} \delta_{AA,1}^{\left(1\right)} & \;=\frac{n}{n+2}\delta_{AA,0}^{\left(1\right)}\\ \text{and}\;\delta_{Aa,0}^{\left(1\right)} & \;=\frac{2}{n+2}\delta_{AA,0}^{\left(1\right)}. \end{array}$$

(In other words, this tells us how to ‘distribute’ the first-order perturbation to *X*_*AA*,0_ over the first-order perturbations to *X*_*AA*,1_ and *X*_*Aa*,0_.)

Substituting equations (A 10), (A 21), (A 22) and (A 23) into equation (A 19), and keeping terms up to order *ϵ*^2^, gives

A 16 $$\begin{array}{r} \begin{array}{rl} -{\dot{\delta }}_{AA,0}^{\left(2\right)} & \;=\frac{2-n-np_{u}}{4n}r_{u}^{n+1}(-2\delta_{AA,1}^{\left(2\right)}+n\delta_{Aa,0}^{\left(2\right)})+\frac{\left(-2+np_{u}\right)}{n}r_{u}^{n+1}\delta_{AA,2}^{\left(2\right)}\\ & \;\;+r_{u}^{n}\left(r_{u}+\frac{\left(\begin{array}{l} v-u-n(2+n^{2}(1-u)-u-v+2n(2-u-v))\\ \;+\;n^{2}(2-n(1-u)-u-v)p_{((2n-1)u+v)/2n} \end{array}\right)}{2n(2n(1-u)-(v-u))}r_{((2n-1)u+v)/2n}\right)\delta_{Aa,1,}^{\left(2\right)}\\ & \;\;-\frac{1}{2}n(1+p_{u})r_{u}^{n+1}\delta_{aa,0}^{\left(2\right)}+\frac{n(3+n)r_{u}^{n+1}}{2{\left(2+n\right)}^{2}}{\left(\delta_{AA,0}^{\left(1\right)}\right)}^{2}\\ {\dot{\delta }}_{AA,1}^{\left(2\right)} & \;=\frac{1}{4}(1+p_{u})r_{u}^{n+1}(-2\delta_{AA,1}^{\left(2\right)}+n\delta_{Aa,0}^{\left(2\right)})-(-1+p_{u})r_{u}^{n+1}\delta_{AA,2}^{\left(2\right)}\\ & \;\;+\frac{\left(n(n(-1+u)+2(-1+v)+(2+n(-1+u)-u-v)p_{((2n-1)u+v)/2n})r_{u}^{n}r_{((2n-1)u+v)/2n}\right)}{2(2n(-1+u)-u+v)}\delta_{Aa,1}^{\left(2\right)}\\ & \;\;+\frac{1}{2}n(1+p_{u})r_{u}^{n+1}\delta_{aa,0}^{\left(2\right)}-\frac{\left(n(1+n)r_{u}^{n+1}\right)}{{\left(2+n\right)}^{2}}{\left(\delta_{AA,0}^{\left(1\right)}\right)}^{2},\\ {\dot{\delta }}_{Aa,0}^{\left(2\right)} & \;=-\frac{r_{u}^{n+1}}{2n}(-2\delta_{AA,1}^{\left(2\right)}+n\delta_{Aa,0}^{\left(2\right)})+\frac{2r_{u}^{n+1}}{n}\delta_{AA,2}^{\left(2\right)},\\ & \;\;+\frac{1}{2}r_{u}^{n}r_{((2n-1)u+v)/2n}\delta_{Aa,1}^{\left(2\right)}+r_{u}^{n+1}\delta_{aa,0}^{\left(2\right)}-\frac{2nr_{u}^{n+1}}{{\left(2+n\right)}^{2}}{\left(\delta_{AA,0}^{\left(1\right)}\right)}^{2},\\ {\dot{\delta }}_{AA,2}^{\left(2\right)} & \;=-r_{u}^{n+1}\delta_{AA,2}^{\left(2\right)}+\frac{(n-1)nr_{u}^{n+1}}{2{\left(2+n\right)}^{2}}{\left(\delta_{AA,0}^{\left(1\right)}\right)}^{2},\\ {\dot{\delta }}_{Aa,1}^{\left(2\right)} & \;=-r_{u}^{n+1}\delta_{Aa,1}^{\left(2\right)}+\frac{2nr_{u}^{n+1}}{{\left(2+n\right)}^{2}}{\left(\delta_{AA,0}^{\left(1\right)}\right)}^{2}\\ \text{and}\;{\dot{\delta }}_{aa,0}^{\left(2\right)} & \;=-r_{u}^{n+1}\delta_{aa,0}^{\left(2\right)}+\frac{r_{u}^{n}r_{((2n-1)u+v)/2n}}{2n}\delta_{Aa,1}^{\left(2\right)}. \end{array} \end{array}$$

Now, each of these equations must be solved.

The equation for ${\dot{\delta }}_{AA,2}^{\left(2\right)}$ can be directly integrated, yielding

A 17 $$\delta_{AA,2}^{\left(2\right)}=\frac{n(n-1)}{2{\left(n+2\right)}^{2}}{\left(\delta_{AA,0}^{\left(1\right)}\right)}^{2}(1-\exp(-r_{u}^{n+1}t)).$$

The same can be done for ${\dot{\delta }}_{Aa,1}^{\left(2\right)}$, yielding

A 18 $$\delta_{Aa,1}^{\left(2\right)}=\frac{2n}{{\left(n+2\right)}^{2}}{\left(\delta_{AA,0}^{\left(1\right)}\right)}^{2}(1-\exp(-r_{u}^{n+1}t).$$

Equation (A 26) can be used to solve for $\delta_{aa,0}^{\left(2\right)}$, yielding

A 19 $$\delta_{aa,0}^{\left(2\right)}=\frac{r_{((2n+1)u+v)/2n}}{{\left(2+n\right)}^{2}r_{u}}{\left(\delta_{AA,0}^{\left(1\right)}\right)}^{2}(1-(1+r_{u}^{n+1}t)\exp(-r_{u}^{n+1}t)).$$

The equations for ${\dot{\delta }}_{AA,1}^{\left(2\right)}$ and ${\dot{\delta }}_{Aa,0}^{\left(2\right)}$ can be manipulated to yield

$$\begin{array}{rl} & \frac{d}{dt}(-2\delta_{AA,1}^{\left(2\right)}+n\delta_{Aa,0}^{\left(2\right)})=-\frac{(2+p_{u})r_{u}^{n+1}}{2}(-2\delta_{AA,1}^{\left(2\right)}+n\delta_{Aa,0}^{\left(2\right)})+2p_{u}r_{u}^{n+1}\delta_{AA,2}^{\left(2\right)}\\ & \;\;-\frac{n(4-u-3v-2(2-n(1-u)-u-v)p_{((2n-1)u+v)/2n})}{2(2n(1-u)-(v-u))}r_{u}^{n}r_{((2n-1)u+v)/2n}\delta_{Aa,1}^{\left(2\right)}\\ & \;\;-np_{u}r_{u}^{n+1}\delta_{aa,0}^{\left(2\right)}+\frac{2n}{{\left(2+n\right)}^{2}}r_{u}^{n+1}{\left(\delta_{AA,0}^{\left(1\right)}\right)}^{2}, \end{array}$$

which can be integrated to give

A 20 $$\begin{array}{rl} & \;-2\delta_{AA,1}^{\left(2\right)}+n\delta_{Aa,0}^{\left(2\right)}\\ & \;\;=\left(\begin{array}{l} \frac{2n(2+(n-1)p_{u})}{{\left(2+n\right)}^{2}(2+p_{u})}-\frac{2np_{u}r_{((2n-1)u+v)/2n}}{{\left(2+n\right)}^{2}(2+p_{u})r_{u}}\\ -\frac{2n^{2}(4-u-3v-2(2-n(1-u)-u-v)p_{((2n-1)u+v)/2n})r_{((2n-1)u+v)/2n}}{{\left(2+n\right)}^{2}(2n(1-u)-(v-u))(2+p_{u})r_{u}} \end{array}\right){\left(\delta_{AA,0}^{\left(1\right)}\right)}^{2}\\ & \;\;+\left(\begin{array}{l} -\frac{2n(r_{u}(n-1-tr_{u}^{n}r_{((2n-1)u+v)/2n})-r_{((2n-1)u+v)/2n})}{{\left(2+n\right)}^{2}r_{u}}\\ -\frac{2n((3n-2)(v-u)+2n(2-n(1-u)-u-v)p_{((2n-1)u+v)/2n})r_{((2n-1)u+v)/2n}}{{\left(2+n\right)}^{2}(2n(1-u)-(v-u))p_{u}r_{u}} \end{array}\right){\left(\delta_{AA,0}^{\left(1\right)}\right)}^{2}\exp(-r_{u}^{n+1}t)\\ & \;\;+\left(\begin{array}{l} \frac{4n(n-2)}{{\left(2+n\right)}^{2}(2+p_{u})}\\ \;+\frac{4n((3n-2)(v-u)+2n(2-n(1-u)-u-v)p_{((2n-1)u+v)/2n})r_{((2n-1)u+v)/2n}}{{\left(2+n\right)}^{2}(2n(1-u)-(v-u))p_{u}(2+p_{u})r_{u}} \end{array}\right){\left(\delta_{AA,0}^{\left(1\right)}\right)}^{2}\\ & \;\;\times \exp\left(-\frac{2+p_{u}}{2}r_{u}^{n+1}t\right). \end{array}$$

We solve for ${\dot{\delta }}_{AA,0}^{\left(2\right)}$ by substituting equations (A 25)–(A 28) into equation (A 24). In doing so, we permit *t* to become relatively large, such that all the time-dependent terms in equations (A 25)–(A 28) approach zero. Accordingly, the sign of ${\dot{\delta }}_{AA,0}^{\left(2\right)}$ tells us that the mutant sterility allele will invade if

$$\lim_{t\to \infty }{\dot{\delta }}_{AA,0}^{\left(2\right)}>0.$$

That is, after substitution and simplification, a recessive sterility allele with penetrance *v* will invade a population monomorphic for sterility with penetrance *u* if

A 21 $$\frac{r_{((2n-1)u+v)/2n}}{r_{u}}>\frac{2(2n(1-u)+u-v)(2+n(1+p_{u}))}{\left(\begin{array}{l} n(8+4n(1-u)-3u-5v)+2(u-v)+(2+n)(2n(1-u)+u-v)p_{u}\\ \;-2n(2-u-v-n(1-u))p_{((2n-1)u+v)/2n} \end{array}\right)}.$$

## Appendix B: Kin-selection analysis

Here, we develop a general model of the evolution of wholly or partly non-reproductive workers using standard kin-selection methodology [38,47]. In this model, a mated queen founds a colony by producing an initial brood of females and/or males. Depending on the model scenario, first-brood females may either mate with first-brood males—from their own or from a different colony—or remain unmated. Then, according to the level of worker sterility *z*, a focal first-brood female (i.e. a worker) invests a proportion of her resources into helping to raise the colony’s next brood—which consists partly of queen-produced offspring (queen-laid females, notated f, and queen-laid males, notated m) and partly of worker-produced offspring (worker-laid females, notated *φ*, and worker-laid males, notated *μ*)—and a proportion of her resources into producing her own offspring. Individuals of the second brood disperse and mate, with each female mating with *n* males, and mated females then found new patches, restarting the cycle.

In this model, we denote a focal worker’s sterility by *Z*, the average sterility on a focal patch by *z* and the average sterility in the population by $\bar{z}$. A focal queen’s sex ratio strategy (investment in females) for her second brood is denoted by *x*, and the average sex ratio strategy among all queens in the population is denoted by $\bar{x}$. The production of queen-laid second-brood females on a focal patch is *f*=*f*(*z*,*x*); the production of queen-laid second-brood males on a focal patch is *m*=*m*(*z*,*x*); the production of worker-laid females by a focal worker is *ϕ*=*ϕ*(*Z*,*z*,*x*); and the production of worker-laid males by a focal worker is *μ*=*μ*(*Z*,*z*,*x*). We denote by $\bar{f}=f(\bar{z},\bar{x})$, $\bar{m}=m(\bar{z},\bar{x})$, $\bar{\phi }=\phi (\bar{z},\bar{z},\bar{x})$, and $\bar{\mu }=\mu (\bar{z},\bar{z},\bar{x})$ the population-average production of each of these four classes, respectively, and by $\tilde{f}=f/\bar{f}$, $\tilde{m}=m/\bar{m}$, $\tilde{\phi }=\phi /\bar{\phi }$ and $\tilde{\mu }=\mu /\bar{\mu }$ the relative production of each of these four classes.

For a gene increasing worker sterility to spread, its carriers, on average, should leave more descendants than other members of the population. Accordingly, natural selection will favour an increase in worker sterility, *z*, when

B 1 $$\frac{\partial \tilde{f}}{\partial z}R_{\mathrm{sis}}+\frac{\partial \tilde{m}}{\partial z}R_{\mathrm{bro}}+\frac{\partial \tilde{\phi }}{\partial Z}R_{\mathrm{dau}}+\frac{\partial \tilde{\phi }}{\partial z}R_{\mathrm{niece}}+\frac{\partial \tilde{\mu }}{\partial Z}R_{\mathrm{son}}+\frac{\partial \tilde{\mu }}{\partial z}R_{\mathrm{neph}}>0.$$

Above, *R*_sis_, *R*_bro_, *R*_dau_, *R*_niece_, *R*_son_ and *R*_neph_ are the (life-for-life) relatedness between a focal female worker and her sister, brother, daughter, niece, son and nephew, respectively, and all derivatives are evaluated at $Z=z=\bar{z}$.

Each term on the left-hand side of condition (B 1) captures how a small increase in worker sterility impacts upon the fitness of different individuals in the population, weighted by the life-for-life relatedness between those individuals and a focal worker, which combines both (i) the reproductive value of those individuals (i.e. their capacity for projecting genes into future generations) and (ii) the extent to which those individuals themselves carry the gene increasing worker sterility. Alternatively, each term can be read as an inclusive-fitness effect experienced by a focal worker who gives up reproduction to become sterile. These interpretations are mathematically equivalent, but we focus on the inclusive-fitness interpretation here, as it is conceptually simpler.

Similarly, natural selection will favour an increase in the queen’s sex allocation strategy (her investment in daughters), *x*, when

B 2 $$\frac{\partial \tilde{f}}{\partial x}R_{\mathrm{dau}\;|\;Q}+\frac{\partial \tilde{m}}{\partial x}R_{\mathrm{son}\;|\;Q}+\frac{\partial \tilde{\phi }}{\partial x}R_{\mathrm{gdau}\;|\;Q}+\frac{\partial \tilde{\mu }}{\partial x}R_{\mathrm{gson}\;|\;Q}>0.$$

Above, *R*_dau | *Q*_ is the relatedness between a focal queen and her daughter, *R*_son | *Q*_ is the relatedness between a focal queen and her son, *R*_*gdau* | *Q*_ is the relatedness between a focal female and her granddaughter (her daughter’s daughter), *R*_*gson* | *Q*_ is the relatedness between a focal female and her grandson (her daughter’s son), and all derivatives are evaluated at $x=\bar{x}$. Each term on the left-hand side of condition (B 2) captures how a small increase in the queen’s investment in daughters, as opposed to sons, impacts upon the fitness of different individuals in the population; alternatively, each term can be read as an inclusive-fitness effect experienced by a focal queen who gives up one of her sons to raise an extra daughter.

For scenario A, the production of queen-laid females, queen-laid males, worker-laid females and worker-laid males is *f*=*xr*_*z*_, *m*=(1−*x*)*r*_*z*_*p*_*z*_, *ϕ*=0 and *μ*=(1−*x*)*r*_*z*_(1−*p*_*z*_)((1−*Z*)/(1−*z*)), respectively. For scenario B, we use *f*=*xr*_*z*_*p*_*z*_, *m*=(1−*x*)*r*_*z*_*p*_*z*_, *ϕ*=0 and *μ*=*r*_*z*_(1−*p*_*z*_)((1−*Z*)/(1−*z*)). For scenario C, we use *f*=*xr*_*z*_*p*_*z*_, *m*=(1−*x*)*r*_*z*_*p*_*z*_, *ϕ*=*yr*_*z*_(1−*p*_*z*_)((1−*Z*)/(1−*z*)) and *μ*=(1−*y*)*r*_*z*_(1−*p*_*z*_)((1−*Z*)/(1−*z*)). And for scenario D, we use *f*=*x*(*z*+*sz*^2^), *m*=(1−*x*)(*z*+*sz*^2^), *ϕ*=*y*(1−*Z*)(1−*c*) and *μ*=(1−*y*)(1−*Z*)(1−*c*). Substituting these definitions into conditions (B 1) and (B 2) recovers conditions (4.3)–(4.8) above.

**B.1. Relatedness calculations**

The life-for-life relatedness of individual A to individual B is *R*_*AB*_=(*F*_*AB*_/*F*_*AA*_)(*c*_*B*_/*c*_*A*_), where *F*_*AB*_ is the consanguinity of individual A and individual B, *F*_*AA*_ is the consanguinity of individual A to herself, *c*_*B*_ is the class reproductive value of individual B and *c*_*A*_ is the class reproductive value of individual A [48]. Note that as individual A is always the same individual within a given condition above, we can instead use *R*_*AB*_=*F*_*AB*_*c*_*B*_ or any multiple thereof without affecting the resulting conditions.

Accordingly, consanguinities needed for the conditions above can be found in table 2. The consanguinities for a female worker under claustral inbreeding are obtained by first calculating the coefficient of inbreeding for a foundress in this mating system (the probability that her two genes at a given locus are identical by descent). Suppose that an offspring is foundress-laid with probability *Q*, and soldier-laid with probability 1−*Q*. If foundress-laid, her coefficient of consanguinity is zero, because patch founders are unrelated. If worker-laid, then her paternally inherited gene comes from her grandmother, and her maternally inherited gene comes, with equal probability, either from her grandfather—who is unrelated to her grandmother—or from her grandmother; in the latter case, her two genes are either copies of the ‘same’ gene in her grandmother, in which case they are identical by descent with probability 1, or are copies of ‘different’ genes from her grandmother, in which case they are identical by descent with probability *G*, where *G* is the offspring’s grandmother’s coefficient of inbreeding. That is, overall, the probability that these two genes are identical by descent is $F=(1-Q)\frac{1}{2}((1+G)/2)$, and at equilibrium, *G*=*F*, which gives *F*=(1−*P*)/(3+*P*). A similar argument gives the same result under diploidy.

**Table 2.** Consanguinities used in inclusive-fitness models.

relationship	notation	haplodiploidy	diploidy
	for outbreeders		
female to daughter	*F*_dau_	$\frac{1}{4}$	$\frac{1}{4}$
female to son	*F*_son_	$\frac{1}{2}$	$\frac{1}{4}$
female to sister	*F*_sis_	$\frac{2+n}{8n}$	$\frac{1+n}{8n}$
female to brother	*F*_bro_	$\frac{1}{4}$	$\frac{1+n}{8n}$
female to niece	*F*_niece_	$\frac{2+n}{16n}$	$\frac{1+n}{16n}$
female to nephew	*F*_neph_	$\frac{2+n}{8n}$	$\frac{1+n}{16n}$
female to daughter’s daughter	*F*_*gdau*_	$\frac{1}{8}$	$\frac{1}{8}$
female to daughter’s son	*F*_*gson*_	$\frac{1}{4}$	$\frac{1}{8}$
	for claustral inbreeders		
female worker to daughter	*F*_dau | *c*_	$\frac{5+Q}{4(3+Q)}$	$\frac{11+Q}{8(3+Q)}$
female worker to son	*F*_son | *c*_	$\frac{1}{2}$	$\frac{11+Q}{8(3+Q)}$
female worker to sister	*F*_sis | *c*_	$\frac{3+2n+Q}{4n(3+Q)}$	$\frac{1+n}{2n(3+Q)}$
female worker to brother	*F*_bro | *c*_	$\frac{1}{3+Q}$	$\frac{1+n}{2n(3+Q)}$
female worker to niece	*F*_niece | *c*_	$\frac{3+6n+Q}{8n(3+Q)}$	$\frac{1+n}{2n(3+Q)}$

**B.2. Class reproductive values**

To determine the class reproductive value of each of the four dispersing offspring classes (queen-laid females, class f; queen-laid males, class m; worker-laid females, class *φ*; and worker-laid males, class *μ*), we first solve for the total reproductive value of all dispersing females, *c*_*F*_=*c*_*f*_+*c*_*φ*_, and the total reproductive value of all males, *c*_*M*_=*c*_*m*_+*c*_*μ*_. Defining $Q=\bar{f}/(\bar{f}+\bar{\phi })$ as the probability that a random dispersing female is queen-laid, and $P=\bar{m}/(\bar{m}+\bar{\mu })$ as the probability that a random male is queen-laid, note that a random female inherits half of her genes from a female in the previous census if she is queen-laid, and three quarters of her genes from a female in the previous census if she is worker-laid; and a random male inherits all his genes from a female in the previous census if he is queen-laid, and half of his genes from a female in the previous census if he is worker-laid. Hence, the recurrence relation *c*_*F*_=(*Q*/2+(3(1−*Q*))/4)*c*_*F*_+(*P*+(1−*P*)/2)*c*_*M*_, with the constraint that *c*_*M*_=1−*c*_*F*_, can be solved to give *c*_*F*_=2(1+*P*)/(3+2*P*+*Q*) and *c*_*M*_=(1+*Q*)/(3+2*P*+*Q*). As an individual’s mating success is not affected by whether they are queen- or worker-laid, we have *c*_*f*_=*Qc*_*F*_, *c*_*φ*_=(1−*Q*)*c*_*F*_, *c*_*m*_=*Pc*_*M*_ and *c*_μ_=(1−*P*)*c*_*M*_, which, overall, gives

$$\begin{array}{rl} c_{f} & \;=\frac{2(1+P)Q}{3+2P+Q},\\ c_{m} & \;=\frac{P(1+Q)}{3+2P+Q},\\ c_{\varphi } & \;=\frac{2(1+P)(1-Q)}{3+2P+Q}\\ \mathrm{and}\;c_{\mu } & \;=\frac{\left(1-P)(1-Q\right)}{3+2P+Q}. \end{array}$$

When all second-brood offspring are queen-laid (*P*=*Q*=1), this yields the expected result that $c_{f}=\frac{2}{3}$, $c_{m}=\frac{1}{3}$, *c*_*φ*_=0 and *c*_*μ*_=0; when all second-brood offspring are worker-laid (*P*=*Q*=0), this yields the expected result that *c*_*f*_=0, *c*_*m*_=0, $c_{\varphi }=\frac{2}{3}$ and $c_{\mu }=\frac{1}{3}$ [49,50].

It is illustrative to examine a special case. When all second-brood females are queen-laid (*Q*=1), this reduces to

$$\begin{array}{rl} c_{f} & \;=\frac{1+P}{2+P},\\ c_{m} & \;=\frac{P}{2+P},\\ c_{\varphi } & \;=0\\ \mathrm{and}\;c_{\mu } & \;=\frac{1-P}{2+P} \end{array}$$

(cf. [45]). In this case, when *P*=1, we have the expected result that the total value of dispersing females is $\frac{2}{3}$ and the total value of males is $\frac{1}{3}$, because of the usual asymmetries of haplodiploidy. But when *P*=0, the total value of dispersing females is $\frac{1}{2}$ and the total value of males is $\frac{1}{2}$. This is because females get half their genes from their mother and half from their father, while males are parthenogenetically produced by worker females, and hence ultimately get half their genes from their mother’s mother and half their genes from their mother’s father. In this way, dispersing females and males have an equal share in producing the next generation of dispersing individuals (cf. [51]).
